# Using Precisely Defined *in vivo* Microbiotas to Understand Microbial Regulation of IgE

**DOI:** 10.3389/fimmu.2019.03107

**Published:** 2020-01-15

**Authors:** Madeleine Wyss, Kirsty Brown, Carolyn A. Thomson, Mia Koegler, Fernanda Terra, Vina Fan, Francesca Ronchi, Dominique Bihan, Ian Lewis, Markus B. Geuking, Kathy D. McCoy

**Affiliations:** ^1^Department of Physiology and Pharmacology, Cumming School of Medicine, Snyder Institute for Chronic Diseases, University of Calgary, Calgary, AB, Canada; ^2^Department of Microbiology, Immunology and Infectious Diseases, Cumming School of Medicine, Snyder Institute for Chronic Diseases, University of Calgary, Calgary, AB, Canada; ^3^Department of Biomedical Research, University of Bern, Bern, Switzerland; ^4^Department of Biological Sciences, University of Calgary, Calgary, AB, Canada

**Keywords:** microbiota, IgE, Tregs, SCFA, gnotobiotic

## Abstract

Early life exposure to microbes plays an important role in immune system development. Germ-free mice, or mice colonized with a low-diversity microbiota, exhibit high serum IgE levels. An increase in microbial richness, providing it occurs in a critical developmental window early in life, leads to inhibition of this hygiene-induced IgE. However, whether this inhibition is dependent solely on certain microbial species, or is an additive effect of microbial richness, remains to be determined. Here we report that mice colonized with a combination of bacterial species with specific characteristics is required to inhibit IgE levels. These defined characteristics include the presence in early life, acetate production and immunogenicity reflected by induction of IgA. Suppression of IgE did not correlate with production of the short chain fatty acids propionate and butyrate, or induction of peripherally induced Tregs in mucosal tissues. Thus, inhibition of IgE induction can be mediated by specific microbes and their associated metabolic pathways and immunogenic properties.

## Introduction

The prevalence of allergies has been increasing over the last 50 years. The hygiene hypothesis postulated a link between decreased microbial exposure and increased type 2 immune responses (1). Later observations revealed that increased hygiene led to changes in both allergic and autoimmune diseases, leading to the counter-regulatory model (2, 3). Growing understanding of the impact of the intestinal microbiome on immune regulation led to formulation of the microflora hypothesis, which suggested that changes in the composition and richness of gut microbial communities underlies allergic diseases (4). Indeed, many studies now support the suggestion that microbial colonization during a critical window in early life is particularly important for development of a regulated immune system in both mice and man (5).

Elevated total serum IgE levels are a hallmark of allergies but are also observed in multiple immunodeficiencies, including some characterized by deficiencies in regulatory T cells (6, 7). We have previously shown that hyper-IgE is associated with an underlying immune dysregulation or deficiency (8). In line with this, germ-free mice display abnormally high serum IgE levels (8–11). We have previously shown that exposure to an increased diversity of microbes in early life, during a “window of opportunity,” could completely inhibit these high serum IgE levels (11). However, it was unclear whether the inhibition of IgE during this critical window was an additive result of increased diversity or rather reflected the presence of a “keystone” microbial species. The underlying cellular and molecular mechanisms involved in inhibition of IgE were also unclear. Regulatory T cells play a major role in suppressing immune responses to self-antigens, commensal microorganisms, and harmless environmental antigens (12). Defects in regulatory T cell induction or function have been associated with the development of autoimmunity, food allergy and age-dependent unbalanced Th2 responses at mucosal sites (7, 13–15). There are thought to be two populations of Tregs; one derived from the thymus, referred to as tTregs, and another originating from naïve T cells in the periphery (pTregs) (16).

Intestinal bacteria, such as some *Clostridia* species, have been shown to induce pTreg differentiation via their production of the short chain fatty acid (SCFA) butyrate in the colon (17, 18). Furthermore, a subpopulation of Tregs, defined as CD4^+^Foxp3^+^RORγt^+^Helios^−^, has been shown to be induced by the microbiota in the small intestine, colon and GALT tissues (19–21). It is unclear, however, whether there is a functional link between microbial colonization, pTreg induction, in particular the RORγt^+^Helios^−^ pTregs subpopulation, and IgE regulation. We hypothesized that certain bacterial species and their production of SCFA could induce the immune regulation of IgE by increasing peripherally induced pTregs. Here, we generated an extensive collection of gnotobiotic mouse colonies and found that three bacterial species that colonize the small intestine in early life can together suppress hygiene-mediated hyper-IgE. We identified acetate production, immunogenicity and mucosa association as key characteristics of bacterial consortia that when combined provide the capacity to inhibit the induction of hyper-IgE.

## Materials and Methods

### Mice, Hygiene Status and, Bacterial Colonization

**Figure 1:**
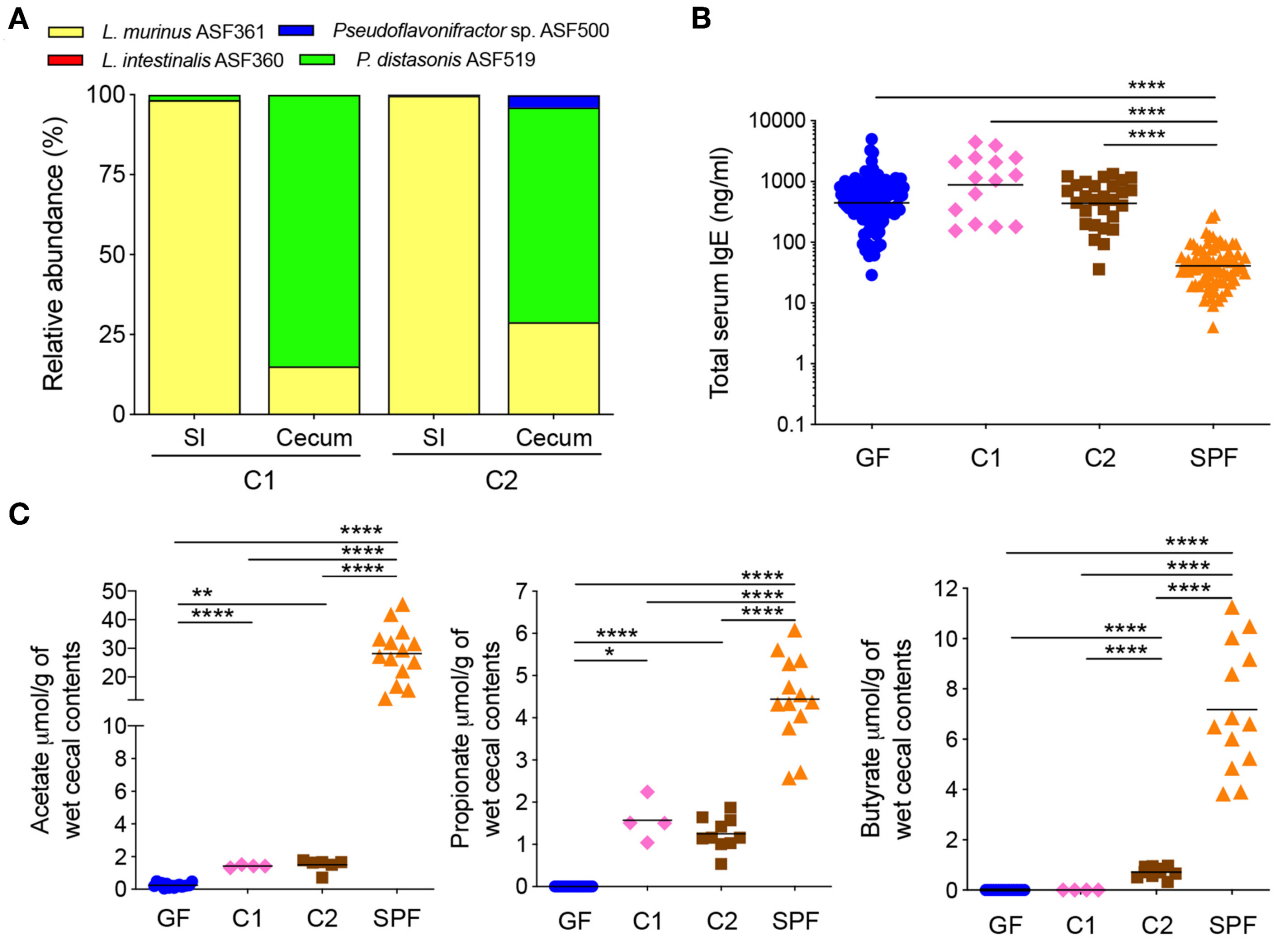
A butyrate-producing bacterial species cannot regulate serum IgE levels **(A)** Relative species abundance in the small intestine (SI) and cecum of a representative C1- and C2-colonized mouse, *n* = 3–4 mice per group. **(B)** Total serum IgE levels in germ-free (GF) (*n* = 113), C1 (*n* = 15), C2 (*n* = 28), and SPF (*n* = 80) mice. **(C)** SCFA levels in the cecal contents of GF (*n* = 15–16), C1 (*n* = 4), C2 (*n* = 6–10), and SPF (*n* = 13–14) mice. Each symbol represents an individual mouse. Black horizontal lines depict the geometric mean **(B)** or mean **(C)**. All mice were 10–13 weeks old. *p < 0.05, **p < 0.01, ****p < 0.0001, calculated by one-way ANOVA with Tukey's **(B)** or Dunnett's **(C)** post-test.

C57BL/6J mice were re-derived to germ-free status via two-cell embryo transfer. Axenic and gnotobiotic mice (germ-free and precisely colonized) were bred and maintained in flexible-film isolators at the Clean Mouse Facility (CMF), University of Bern, Switzerland or at the International Microbiome Centre (IMC), University of Calgary, Canada. The germ-free and gnotobiotic C57BL/6J strain used in Bern and Calgary were identical. For some experiments, mice were housed in individually ventilated isocages (Tecniplast) in the IMC. Germ-free status was routinely monitored by culture-dependent and—independent methods and all germ-free colonies were independently confirmed to be pathogen-free. To start gnotobiotic mouse colonies, germ-free breeding pairs were gavaged with single or mixed bacterial cultures, as indicated. Gram staining, SYTOX^TM^ (Fisher) DNA staining and 16S rRNA gene amplicon sequencing of their intestinal contents were performed to confirm their colonization status. The offspring of these gnotobiotic breeding pairs were analyzed at 10–13 weeks of age, unless stated otherwise. SPF mice were either purchased from Envigo (Switzerland) or bred and maintained at the University of Calgary. The SPF mice at the University of Calgary were fed the same autoclaved diet as their germ-free and gnotobiotic counterparts. Although two different SPF colonies were used, one in Switzerland (Envigo, Netherlands) and another in Canada (in house SPF colony) we did not observe any significant differences in our readouts between these two SPF colonies. The only data derived from Envigo SPF mice are *n* = 18 of a total of *n* = 80 mice shown in Figure 1B as well as the data shown in Supplementary Figure 3. All animal experiments were in accordance with the guidelines established by either the Swiss Federal Veterinary Office or by the Canadian Council for Animal Care and were approved by the Commission for Animal Experimentation of the Veterinary Office of Canton Bern or by the University of Calgary Animal Care Committee.

### Bacterial Culture

Bacteria were cultured in Brain Heart Infusion (BHI) medium as previously described (22). Bottles and media were gassed with 10% H_2_, 10% CO_2_, and 80% N_2_ while in a Whitley A95 anaerobic incubator before being sealed and inoculated with specific bacterial species.

### Bacterial Flow Cytometry

Bacterial flow cytometry was performed as described previously (23, 24). Briefly, bacteria were cultured for 24–48 h as described above, with the modification that the BHI media was filter-sterilized before inoculation. For growing *Akkermansia muciniphila* YL44, 0.25 g/L of autoclaved Type II hog gastric mucin (Sigma) was added to the filter-sterilized BHI media. Bacterial cultures were centrifuged for 10 min at 3,000 *g*, washed twice and re-suspended in sterile-filtered PBS/2%BSA/0.02% sodium azide and diluted to ~10^7^ bacteria/ml (OD_595_ 0.1 = 10^8^ bacteria/ml). Serum was diluted 1:10 in PBS/2%BSA/0.02% sodium azide, heat-inactivated at 56°C for 30 min and centrifuged at 16,000 *g* for 5 min at 4°C to remove any bacteria sized contaminants. This serum supernatant was used to perform serial dilutions. 2.5 × 10^5^ bacteria were added to each well and incubated with the serial dilutions of serum for 1 h at 4°C, centrifuged for 10 min at 3,000 *g*, washed twice and then re-suspended in monoclonal APC rat anti-mouse IgG1 (1:40, A85-1) (BD) and BV605 rat anti-mouse IgA (1:40, C10-1) (BD) and incubated overnight at 4°C. The bacteria were washed twice in PBS/2%BSA/0.02% sodium azide and then re-suspended in PBS/2%BSA/0.02% sodium azide and acquired on a FACS Canto (BD) using FSc (forward scatter) and SSc (side scatter) in logarithmic mode. Data was analyzed using FlowJo software (Tree Star Inc.).

### Small Intestinal Wash Collection

The entire small intestine was removed and washed with 5 ml of ice-cold intestinal wash buffer [10% 0.5 M EDTA pH 8.0, 10% 10x PBS, ddH_2_O and 0.1 mg ml^−1^ soybean trypsin inhibitor (Sigma)] with 40 μl of 100 μM phenylmethylsulfonylfluoride (PMSF, Sigma) per 5 ml of intestinal wash buffer. The small intestinal wash was centrifuged at 4,000 *g* for 10 min at 4°C and stored at −80°C.

### DNA Extraction From Intestinal Contents

Small intestines, ceca and colons were opened longitudinally. Contents were removed and snap-frozen in liquid nitrogen and stored at −80°C. DNA was extracted from the contents using the QIAamp Fast DNA Stool Mini Kit (Qiagen), according to the manufacturer's instructions. Briefly, samples were homogenized in InhibitEX buffer by bead-beating using differentially sized beads (glass beads, 0.5–0.75 mm; zirconia beads, <100 μM), treated with lysis buffer (1.2% Triton X-100, 2 mM EDTA, 20 mM Tris·HCl) containing lysozyme (20 mg ml^−1^, Sigma), followed by Proteinase K treatment. Bacterial DNA was ethanol-precipitated on a column membrane and eluted with sterile water. Procedural blank DNA extractions were performed in parallel with each batch of DNA extractions and run as controls during the downstream PCR steps to confirm the absence of contaminating DNA sequences from the extraction process.

### 16S rRNA Gene Amplicon Sequencing

Two platforms were used for 16S rRNA gene amplicon sequencing.

For the gnotobiotic models C1 and C2 (Supplementary Table 1) the 16S rRNA gene segments spanning the variable V5 and V6 regions were amplified from DNA from intestinal content samples using a multiplex approach with the barcoded forward fusion primer 5′-*CCATCTCATCCCTGCGTGTCTCCGACTCAG* BARCODE ATTAGAT ACCCYGGTAGTCC-3′ in combination with the reverse fusion primer 5′-*CCTCT CTATGGGCAGTCGGTGATACGAGCTGACGACARCCATG*-3′. The sequences in *italics* are Ion torrent PGM-specific adaptor sequences. The PCR amplified 16S rRNA V5-V6 amplicons were purified and prepared for sequencing on the Ion torrent PGM system according to the manufacturer's instructions (Life Technologies). Samples with over 1,000 reads were used for the analysis. Data analysis was performed using the QIIME pipeline version 1.8.0 (25). Operational taxonomic units were picked using UCLUST (26) with a 97% sequence identity threshold followed by taxonomy assignment using a custom C1 or C2 database for gnotobiotic C1 and C2 intestinal samples. For all other gnotobiotic models, the 16S rRNA gene segment spanning the variable V4 region was amplified from DNA extracted from intestinal content samples using a multiplex approach with the barcoded forward fusion primer 5′- AATGATACGGCGACCACCGAGATCTACAC i5BARCODE TATGGTAATTGTGTGCCAGCMGCCGCGGTAA-3′ in combination with the reverse fusion primer 5′CAAGCAGAAGACGGCATACGAGAT i7BARCODE AGTCAGTCAGCCGGACTACHVGGGTWTCTAAT-3′. One PCR reaction contained 0.25 mM of each primer, KAPA HiFi Hot Start Ready Mix (Roche) and 20–100 μg of template DNA. PCR conditions were 98°C for 2 min, followed by 25 cycles of 98°C for 30 s, 55°C for 30 s and 72°C for 20 s and a final extension step at 72°C for 7 min. Amplification of the V4 gene region was verified by electrophoresis on a 1.2% agarose gel. Reactions were purified using NucleoMag NGS clean-up beads (Macherey-Nagel) and were normalized using the SequalPrep Normalization Plate Kit (Invitrogen). Amplicons were pooled and concentration and quality were determined using the Qubit HS DNA kit (Qubit) and the Tapestation D1000 assay (Agilent), respectively. Amplicon sequencing was done on a MiSeq Benchtop DNA sequencer (Illumina) using a V2-500 cycle kit (Illumina Inc.). Using the Dada2 (27), the phyloseq (28) and the reshape2 packages (29) within R (30), forward and reverse reads were trimmed to 230 and 210 bp, respectively, and chimeras were removed using the removeBimeraDenovo function. Taxonomy was assigned using custom databases containing the 16S rRNA gene sequences of all the bacterial species used in the gnotobiotic models. Amplicon sequence variants (ASVs) that were present at <0.5% were excluded.

### 16S rRNA Gene Full Length Sequencing

To confirm monocolonization status feces were collected from monocolonized mice and DNA was extracted. 16S rRNA full length PCR was performed using forward primers fD1 5′-AGA GTT TGA TCC TGG CTC AG-3′ and fD2 5′-AGA GTT TGA TCA TGG CTC AG-3′ and the reverse primer rP1 5′ACG GTT ACC TTG TTA CGA CTT-3′ (31). Each PCR reaction contained 0.2 μM of each primer, 1X Green GoTaq® Flexi buffer (Promega), 0.2 mM dNTP (Promega), 1.25 Units of GoTaq® DNA polymerase (Promega) and 100 ng of extracted fecal DNA. PCR conditions were 94°C for 5 min, followed by 35 cycles of 94°C for 1 min, 43°C for 1 min and 72°C for 2 min and a final extension step at 72°C for 7 min. PCR products were submitted for Sanger sequencing and sequencing results were blasted against the NCBI blast database.

### Cell Isolation and Intracellular Cytokine Staining

The entire small intestine and the entire colon were excised and the residual fat and Peyer's patches (PP) were removed. The intestines were opened longitudinally, rinsed in Dulbecco's Phosphate-Buffered Saline (DPBS) lacking CaCl_2_ and MgCl_2_ (Invitrogen), cut into 1-2 cm long pieces and washed once (colon) or twice (small intestine) in 25 ml of Hank's balanced salt solution (HBSS) containing 0.5 mM EDTA, 10 mM HEPES and 5% Horse serum (HS) for 20 min at 37°C with shaking at 220 rpm in order to remove the epithelial layer. Here HBSS consisted of 5.4 mM KCl, 0.3 mM NaH_2_PO_4_, 0.4 mM KH_2_PO_4_ and 137 mM NaCl. Residual tissue was rinsed with DPBS and transferred into HBS/5% HS solution (HBSS with 10 mM HEPES and 25 mM NaHCO_3_) containing 1.0 mg ml^−1^ collagenase Type VIII (Sigma) and 10 U DNAse I (Roche) and minced and digested for 20–30 min (small intestine) or 25–35 min (colon) at 37°C with shaking at 220 rpm. The resulting cell suspension was passed through a cell strainer (100 μM). Cells were washed with HBS/5%HS and centrifuged at 400 *g* for 5 min and re-suspended for quantification.

For Figure 3, after digestion and centrifugation the small intestine cell pellet was re-suspended in 40% Percoll solution and layered on top of a 70% Percoll solution (GE Healthcare). Gradient centrifugation was carried out at 700 *g* for 20 min at room temperature with no break. Cells lying at the 40%/70% interphase were collected and washed with HBS/5% HS and centrifuged at 400 *g* for 5 min and re-suspended for quantification. Mesenteric lymph nodes (MLNs) and PP were digested with HBS/5%HS containing 1 mg ml^−1^ collagenase IA (Sigma) and 10 U DNAse I (Roche) for 20 min at 37°C and passed through a cell strainer (100 μM), washed with HBS/5%HS and centrifuged at 400 *g* for 5 min and re-suspended for quantification. Spleens were passed through a cell strainer (100 μM) and centrifuged at 400 *g* for 5 min. Spleen cell pellets were re-suspended in 1 ml of 0.88% NH_4_Cl for 10 min at room temperature to lyse red blood cells. Cells were washed with HBS/5%HS and centrifuged at 400 *g* for 5 min and re-suspended for quantification.

For intracellular cytokine staining small intestinal single cell suspensions were stimulated for 4 h at 37°C/5% CO_2_ in Iscove's Modified Dulbecco's Media (IMDM, Fisher) containing 10% Fetal Bovine Serum (FBS, Fisher) in the presence or absence of 50 ng ml^−1^ phorbol 12-myristate 13-acetate (PMA, Sigma) and 750 ng ml^−1^ ionomycin (Invitrogen) with 10 μg ml^−1^ Brefeldin A (Sigma).

### Flow Cytometry

Cells were washed twice with DPBS and stained with either fixable viability dye eFluor506 (eBioscience) or fixable viability stain 780 (BD) and anti-CD16/CD32 (2.4G2; BD) in DPBS for 10 min at 4°C. Cells were then washed with FACS buffer containing 2% fetal calf serum (Invitrogen), 2.5 mM EDTA (MP Biomedicals) in PBS. For cell surface staining, cell pellets were re-suspended in antibodies diluted in FACS buffer and stained for 30 min at 4°C. Intra-nuclear staining was performed using the Foxp3 staining kit (eBioscience). Intracellular cytokine staining was performed with the Cytofix/Cytoperm staining kit (BD). The following mouse-specific conjugated antibodies were used: CD45-PerCP-Cy5.5 (30-F11; BD), CD45-BV510 (30-F11; BD), TCRβ-BV786 (H57-597; BD), CD4-Pe-Cy7 (RM4-5; BD), Helios-AlexaFluor488 (22F6; BD), CD3-PB (145-2C11; Biolegend), CD4-BV785 (RM4-5; Biolegend), Helios-FITC (22F6; Biolegend), IL10-Pe-Cy7 (JES5-16E3; Biolegend) Foxp3-AlexaFluor700 (FJK-16s; eBioscience), and RORγt-PE (Q31-378; eBioscience). Cells were acquired on a FACS Fortessa (BD Biosciences) or a FACS Canto (BD) with FACSDIVA software (BD). Data analysis was performed using FlowJo software (Tree Star Inc.).

### IgE ELISA and IgE Electrochemiluminescence Immunoassay (IgE-ECL)

Blood was collected in serum-separating tubes (Sarstedt and BD) and total serum IgE concentrations were measured using either the BD OptEIA Mouse IgE ELISA Set (BD) according to the manufacturer's instructions, or a multi-array (Meso Scale Discovery) utilizing the BD OptEIA Mouse IgE ELISA Set.

### IgA and IgG1 Electrochemiluminescence Immunoassay

Total concentrations of IgA and IgG1 in serum and small intestinal wash were determined using the Mouse Isotyping Panel 1 kit according to the manufacturer's instructions (Meso Scale Discovery).

### LC/MS/MS Based Short Chain Fatty Acid Metabolite Analysis

Cecal contents were collected, snap-frozen in liquid nitrogen and stored at −80°C until processed. Native SCFAs were extracted from cecal samples with an ice-cold 50% acetonitrile solution (2:1 (v/w) ratio) containing known amounts of the following ^13^C-SCFA analytical standards used as internal standards (IS): acetic acid (1,2-13C2, 99%, #CLM-113, Cambridge Isotope Laboratories, Inc, Andover, MA, USA), Propionic acid (13C3, 99%, #589586, Sigma-Aldrich, St. Louis, MO, USA) and Butyric acid (1,2-13C2, 99%, #491993, Sigma-Aldrich) (Supplementary Table 2). Samples were homogenized at 30 Hz for 3 min with a tissue lyzer (Qiagen) and centrifuged at 18,000 *g* for 10 min at 4°C. Supernatants were collected and centrifuged under the same conditions. Supernatants were then derivatized with EDC (N-(3-Dimethylaminopropyl)-N′-ethylcarbodiimide hydrochloride) and aniline (32, 33) as follows: 100 μL aliquot were incubated with EDC (50 mM, final concentration) and aniline (100 mM, final concentration) for 2 h at 4°C. An aliquot of each sample was further diluted with 50% HPLC-grade methanol/water. All samples were stored at 4°C until analysis on the same day.

LC-MS/MS analysis of cecal contents was performed on a Vanquish^TM^ UHPLC System coupled to a TSQ Quantum^TM^ Access MAX triple quadrupole Mass Spectrometer (Thermo Scientific) equipped with an electrospray ionization (HESI-II) probe. The UHPLC-MS platform was controlled by Xcalibur^TM^ data system (Thermo Scientific).

Chromatographic separation was achieved on a Hypersil GOLD^TM^ C18 column (200 X 2.1 mm, 1.9 μm, Thermo Scientific) using a binary solvent system composed of LC-MS grade H_2_O with 0.1% (%v/v) formic acid (Solvent A) and LC-MS grade methanol with 0.1% (%v/v) formic acid (solvent B). The following 21 min gradient was used: 0–1 min, 40%B; 1–7 min, 40–98%B; 7–15 min, 98%B; 15–16 min, 98–40%B; 16–21 min, 40%B. LC eluent was diverted to waste for the first 5 min of the run. The flow rate was 200 μL/min and the sample injection volume 2 μL. The auto sampler was kept at 4°C and the column at 30°C.

MS/MS data were acquired in positive ionization mode with the mass spectrometer operating in Selected Reaction Monitoring (SRM) mode. Fragmentation parameters were optimized using the EZ Tune program with direct infusion of the derivatized analytical grade standards. Subsequently, the following transitions, corresponding to the three derivatized native SCFAs and respective derivatized ^13^C-SCFA standards, were monitored, with a scan time of 0.05 s and a fixed collision energy of 14 eV: [M+H]^+^ *m/z* 136.07, 138.08, 150.09, 153.10, 164.10, 166.11 → *m/z* 94.06. Electrospray ionization source conditions were as follows: spray voltage of 3,000 V, vaporizer temperature of 300°C, sheath gas of 5 (arbitrary units), sweep gas of 1 (arbitrary units), auxiliary gas of 2 (arbitrary units), capillary temperature of 275°C.

Data analyses, on the converted mzXML files, were conducted in MAVEN (34, 35). In short, for each SCFA, the determination of the native SCFA concentration was based on the ^12^C/^13^C signal intensity ratio and the respective ^13^C-IS concentration.

### Statistical Analysis

Statistical analysis was performed by one-way ANOVA with Tukey's or Dunnett's post-test depending on whether the comparison was performed between all means (Tukey's) or compared to a control mean (Dunnett's) using GraphPad Prism 8 (GraphPad Software Inc.). The data for serum IgE concentrations was first log transformed before performing one-way ANOVA with Tukey's post-test. The SCFA in colon vs. cecum (in Supplementary Figure 1) were compared using Student's *t*-test. A *p* < 0.05 was considered statistically significant. *P*-values are indicated as follows: ^*^p < 0.05, ^**^p < 0.01, ^***^p < 0.001, ^****^p < 0.0001, not significant (ns).

## Results

### The Presence of a Butyrate-Producing Bacterial Species Has a Minimal Effect on Adult Serum IgE Levels

We have previously shown that mice colonized with the Altered Schaedler Flora (ASF), harboring eight commensal intestinal bacterial species (36), display a dichotomy in terms of development of high IgE levels (11). High IgE levels in adulthood correlated with a low ASF diversity early in life while a high ASF diversity early in life suppressed induction of IgE. We also noted that the presence of the butyrate-producing bacteria *Pseudoflavonifractor* sp. ASF500 early in life correlated with suppression of IgE (11). We and others have also previously shown that ASF colonization (37) or colonization with microbial consortia containing *Clostridia* species (38, 39) results in the induction of intestinal pTregs and the SCFA butyrate has been demonstrated to support pTreg induction (17, 18). To test whether butyrate produced by *Pseudoflavonifractor* sp. ASF500 could suppress hyper-IgE, we gavaged germ-free C57BL/6 breeding pairs with either microbial Community #1 (C1, Supplementary Table 1) consisting of three non-butyrate-producing ASF species we know do not suppress hyper-IgE, or community C2, which had the butyrate-producer *Pseudoflavonifractor* sp. ASF500 added to the community (Supplementary Table 1). This approach was necessary because *Pseudoflavonifractor* sp. ASF500 is unable to colonize a germ-free mouse on its own and requires the presence of other bacterial species (data not shown). Colonization with either C1 or C2 in the small intestine and cecum of the offspring was confirmed by 16S rRNA amplicon sequencing (Figure 1A). *Pseudoflavonifractor* sp. ASF500 was only detected in the C2 group and *Lactobacillus intestinalis* ASF360 (present in the inoculum for C1 and C2) was below the detection limit in both groups.

Total serum IgE levels were measured in large control cohorts of GF and specific pathogen free (SPF) mice (Figure 1B). As previously shown (11), IgE levels in adult GF mice were elevated and significantly increased above SPF levels. We found that IgE levels in adult C1- or C2-colonized offspring remained significantly elevated at the level of GF controls (Figure 1B). We found that colonization with both C1 and C2 led to significant increases in acetate and propionate levels compared to GF levels, whereas only C2 significantly increased butyrate levels in the cecal contents, as expected (Figure 1C). All SCFA levels in the C1 and C2 cohorts remained significantly lower than that found in SPF mice (Figure 1C). These data indicated that low levels of SCFA were insufficient to inhibit hygiene-induced hyper-IgE. Of note, SCFA levels in cecal contents were comparable to the levels observed in colon contents (Supplementary Figure 1).

### A Further Increase in Microbial Diversity Does Not Inhibit Hyper-IgE Despite Induction of pTregs

**Figure 2:**
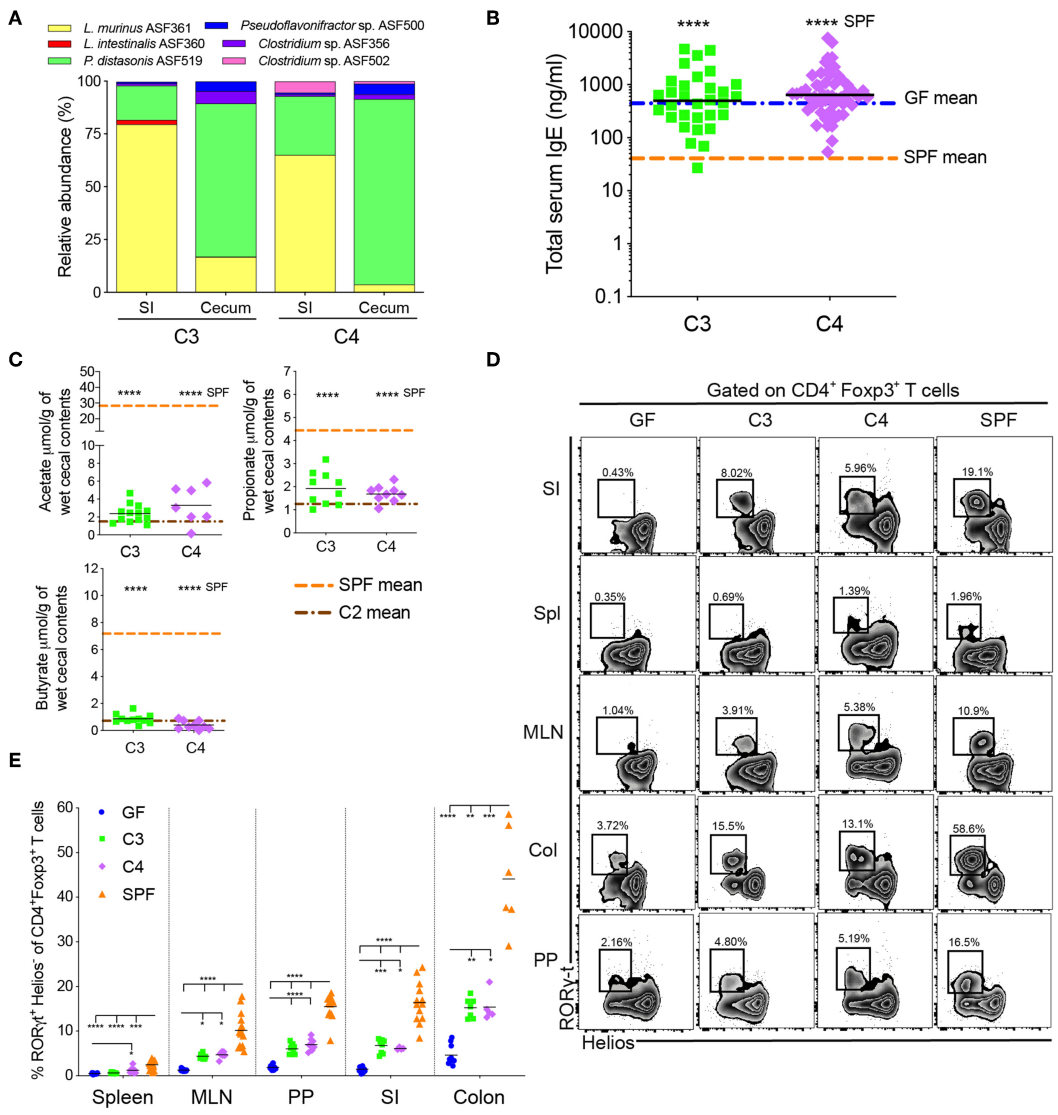
An increase in microbial diversity does not inhibit hyper-IgE despite pTreg induction. **(A)** Relative species abundance in the small intestine (SI) and cecum of a representative C3- and C4-colonized mouse, *n* = 3 mice per group. **(B)** Total serum IgE levels in C3 (*n* = 31) and C4 (*n* = 56) mice. The blue and orange horizontal lines represent the geometric mean of the GF and SPF cohorts, respectively (from Figure 1B). **(C)** SCFA levels in the cecal contents of C3 (*n* = 10–13) and C4 (*n* = 7–9) mice. The orange and brown horizontal lines represent the mean of the SPF and C2 cohorts, respectively (from Figure 1C). **(D)** Representative flow cytometry plots of RORγt^+^Helios^−^ Tregs in different tissues (Spl, spleen; PP, Peyer's patches; MLN, mesenteric lymph nodes; SI, small intestinal lamina propria; Col, colon lamina propria) of GF, C3, C4 and SPF mice. **(E)** Frequencies of RORγt^+^Helios^−^ Tregs among CD45^+^TCRβ^+^CD4^+^Foxp3^+^ T cells in different tissues of GF (*n* = 10–15), C3 (*n* = 4–9), C4 (*n* = 4–8), and SPF (*n* = 7–16) mice. All mice were 10–13 weeks old. SCFA and RORγt^+^Helios^−^ Treg data are pooled from at least two independent experiments. Each symbol represents an individual mouse. Black horizontal lines depict the geometric mean **(B)** or mean **(C,E)**. *p < 0.05, **p < 0.01, ***p < 0.001, ****p < 0.0001, calculated by one-way ANOVA with Tukey's **(B,E)** or Dunnett's **(C)** post-test.

We next investigated whether sequentially increasing bacterial diversity would lead to suppression of hyper-IgE. We therefore added *Clostridium* sp. ASF356 to the C2 community to form community C3 and added *Clostridium* sp. ASF502 to C3 to generate C4 (Supplementary Table 1). 16S rRNA amplicon sequencing of small intestinal and cecal contents from adult C3- and C4-colonized offspring confirmed the presence of *Clostridium* sp. ASF356 in both C3- and C4-colonized mice and *Clostridium* sp. ASF502 only in C4-colonized mice (Figure 2A). Despite the increase in microbial richness, serum IgE levels in C3- and C4-colonized mice remained comparable to GF levels and significantly elevated compared to SPF IgE levels (Figure 2B). Furthermore, despite the increase in microbial richness, SCFA levels in cecal content from C3- and C4-colonized mice did not differ from those found in C2-colonized mice (Figure 2C). Since regulatory T cells are thought to be important in regulating Type 2 immunity (19), we next assessed induction of Tregs (gating strategy shown in Supplementary Figure 2A). We found that although there was only a minimal effect on the proportions and total numbers of Foxp3^+^ Tregs (Supplementary Figures 2B–D), colonization with both C3 and C4 led to significant induction of RORγt^+^Helios^−^ pTregs in the mesenteric lymph nodes (MLN), Peyer's patches (PP), small intestine (siLP) and colon lamina propria (cLP) (Figures 2D,E). C4 colonization also led to an increase of RORγt^+^Helios^−^ pTregs in the spleen (Figures 2D,E). The transcription factor Helios has been suggested to identify tTregs whereas pTregs are characterized as Helios negative (40, 41). However, it remains controversial whether Helios, or other markers such as Neuropilin-1 (42, 43), can unequivocally identify tTregs vs. pTregs (44). We have therefore included expression of RORγt to better identify microbially-induced pTreg (19–21). These results indicate that although the microbial communities of C3 and C4 produced SCFA and induced RORγt^+^Helios^−^ pTregs, this was not sufficient to inhibit hyper-IgE.

### A More Diverse Microbial Community Suppresses Hyper-IgE

**Figure 3:**
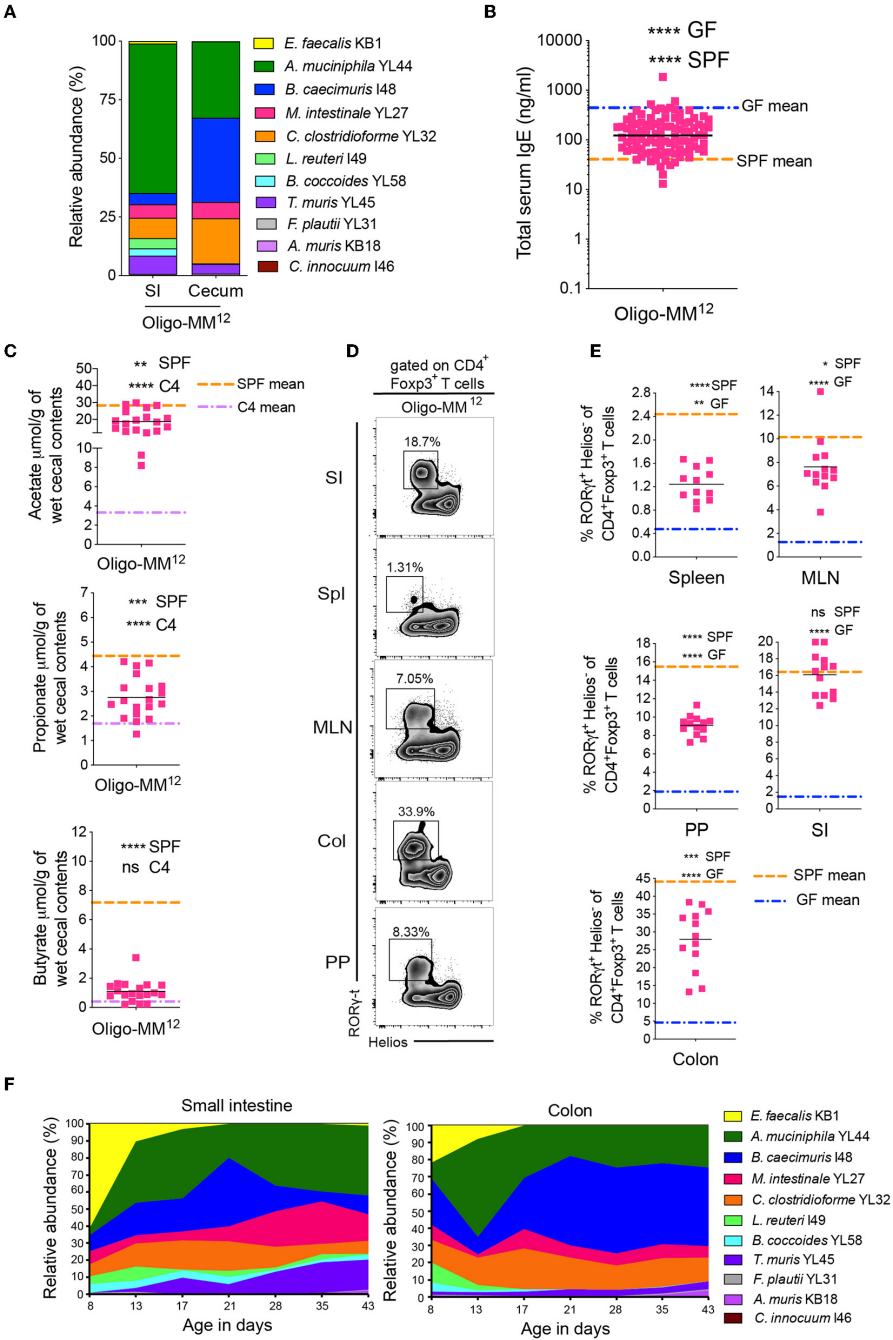
A further increase in microbial diversity suppresses hyper-IgE and demonstrates a dynamic bacterial colonization profile in the small intestine and colon in early life. **(A)** Relative species abundance in the small intestine (SI) and cecum of a representative adult Oligo-MM^12^ mouse, *n* = 4 mice. **(B)** Total serum IgE levels in Oligo-MM^12^ (*n* = 100) mice. The blue and orange horizontal lines represent the geometric mean of the serum IgE levels from the GF and SPF cohorts, respectively (from Figure 1B). **(C)** SCFA levels in the cecal contents of Oligo-MM^12^ (*n* = 19) mice. The orange and purple horizontal lines represent the mean SCFA levels in the SPF and C4 cohorts, respectively (from Figures 1C, 2C). **(D)** Representative flow cytometry plots of RORγt^+^Helios^−^ Tregs in different tissues (Spl, spleen; PP, Peyer's patches; MLN, mesenteric lymph nodes; SI, small intestinal lamina propria; Col, colon lamina propria) of Oligo-MM^12^ mice. **(E)** Frequencies of RORγt^+^Helios^−^ Tregs among CD3ε^+^CD4^+^Foxp3^+^ T cells in different tissues of Oligo-MM^12^ (*n* = 12–13) mice. The blue and orange horizontal lines represent the mean of the GF and SPF cohorts, respectively (from Figure 2E). **(F)** Relative species abundances of Oligo-MM^12^ bacterial species in the small intestine and colon of 8 to 43-day old Oligo-MM^12^ mice. *n* = 2–5 mice per time point. All mice were 10–13 weeks old **(A–E)**. SCFA and RORγt^+^Helios^−^ Treg data are pooled from at least two independent experiments. Each symbol represents an individual mouse. Black horizontal lines depict the geometric mean **(B)** or mean **(C,E)**. *p < 0.05, **p < 0.01, ***p < 0.001, ****p < 0.0001, calculated by one-way ANOVA with Tukey's **(B,E)** or Dunnett's **(C)** post-test.

We have previously demonstrated that bacterial diversity during early life is important to suppress hyper-IgE (11). Since our sequential increase of ASF diversity (C1 < C2 < C3 < C4) had no inhibitory effect on serum IgE levels, we hypothesized that none of these microbiotas (C1 to C4) reach a high enough diversity early in life to suppress hyper-IgE. Since the full ASF showed only partial suppression of hyper-IgE depending on colonization diversity early in life (11) we hypothesized that further experimentally increasing the microbial diversity will eventually result in suppression of hyper-IgE. We recently established a gnotobiotic microbiota consisting of 12 defined and well-characterized bacterial species. This microbiota is referred to as either stable Defined Moderately Diverse Microbiota from mice (sDMDMm2) (45, 46) or Oligo-mouse microbiota (Oligo-MM^12^) (22, 47) (Supplementary Table 3). Analysis of the microbiota composition in the small intestine and cecum of our gnotobiotic Oligo-MM^12^ mouse cohort revealed that 11 of the 12 species were detectable by 16S rRNA amplicon sequencing (Figure 3A) with *Bifidobacterium longum* subsp. *animalis* YL2 undetectable [as has previously been observed (22)]. More importantly, however, colonization with the Oligo-MM^12^ resulted in suppression of hyper-IgE (Figure 3B), which correlated with increased production of acetate and propionate compared to C4-colonized mice while butyrate levels remained unchanged (Figure 3C). However, the suppression of hyper-IgE did not reach the levels observed in SPF mice (Figure 2B), which indicates that increasing bacterial diversity even further to SPF enhances suppression of hygiene-induced hyper-IgE (11). Notably, colonization with Oligo-MM^12^ also resulted in significant induction of RORγt^+^Helios^−^ pTregs (Figures 3D,E and Supplementary Figure 3).

We next followed the Oligo-MM^12^ colonization dynamics in early life in the small intestine and colon starting from day 8 until day 43 after birth (Figure 3F). Up until about 2 weeks of age there was a dominance of *Enterococcus faecalis* KB1 and *Akkermansia muciniphila* YL44 in both the small intestine and colon. The level of *E. faecalis* KB1 then declined drastically concomitantly with an increase in *Bacteroides caecimuris* I48 (Figure 3F). These changes coincided with weaning of the pups, suggesting that the change in diet from milk to solid food contributes to the increase of certain bacterial species. In light of our previous findings (11), these data suggest that the two species (*E. faecalis* KB1 and *A. muciniphila* YL44) most prominent in early life may contribute to regulation of IgE levels. In addition, since Clostridia species are known to be dominant producers of SCFA, and we found that *C. clostridioforme* YL32 and Clostridiales species *B. coccoides* YL58, were also present in early life, albeit at lower levels, we further dissected the protective potential of these four bacterial species.

### Two Dominant Early Life Clostridiales Are Unable to Regulate Serum IgE Levels

**Figure 4:**
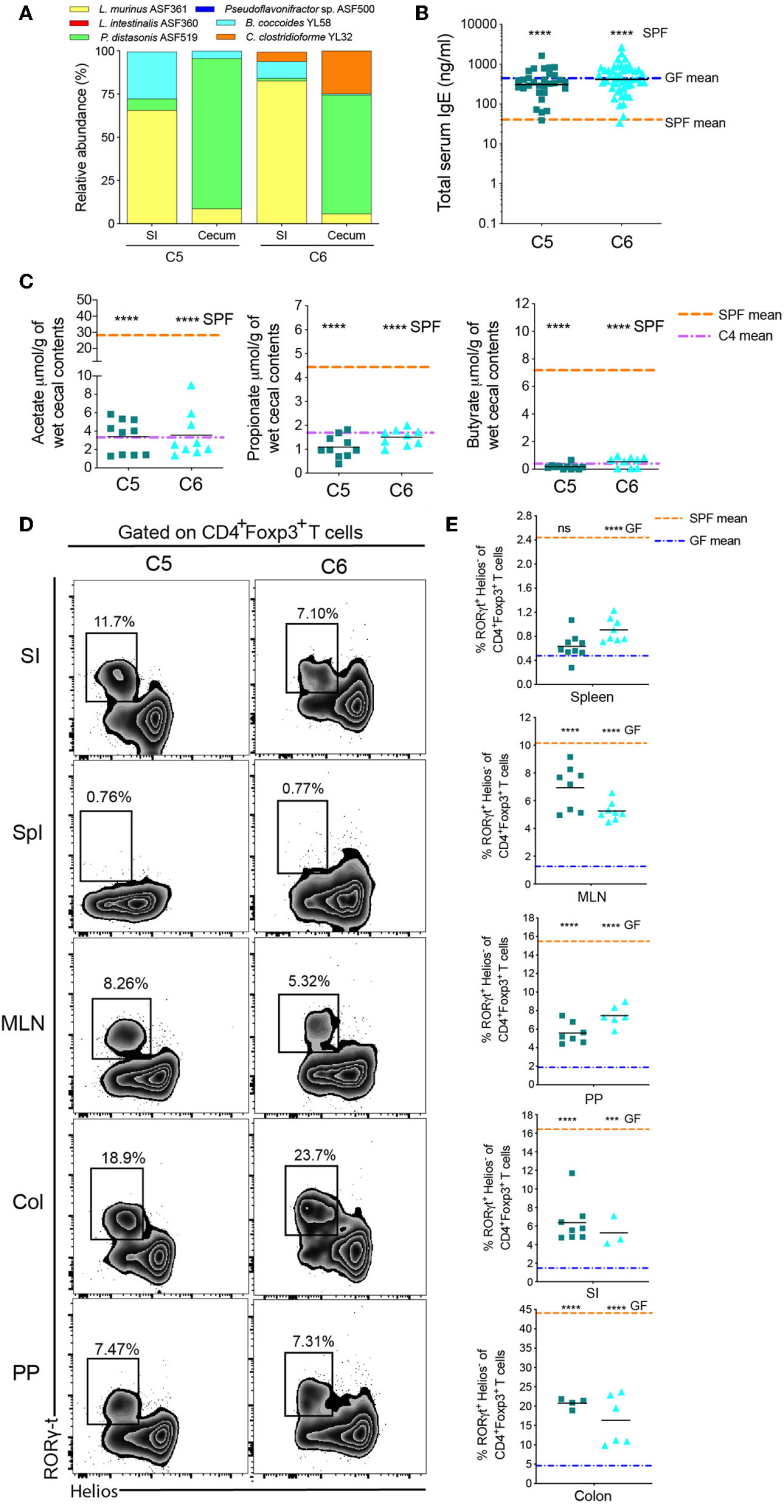
Two early-life Oligo-MM^12^ Clostridiales bacteria are unable to regulate serum IgE levels. **(A)** Relative species abundance in the small intestine (SI) and cecum of a representative C5- and C6-colonized mouse, *n* = 2–4 mice per group. **(B)** Total serum IgE levels in C5 (*n* = 30) and C6 (*n* = 49) mice. The blue and orange horizontal lines represent the geometric mean of the serum IgE levels from the GF and SPF cohorts, respectively (from Figure 1B). **(C)** SCFA levels in the cecal contents of C5 (*n* = 10–11) and C6 (*n* = 9) mice. The orange and purple horizontal lines represent the mean SCFA levels in the SPF and C4 cohorts, respectively (from Figure 1C, 2C). **(D)** Representative flow cytometry plots of RORγt^+^Helios^−^ Tregs in different tissues (Spl, spleen; PP, Peyer's patches; MLN, mesenteric lymph nodes; SI, small intestinal lamina propria; Col, colon lamina propria) of C5 and C6 mice. **(E)** Frequencies of RORγt^+^Helios^−^ Tregs among CD45^+^TCRβ^+^CD4^+^Foxp3^+^ cells in different tissues of C5 (*n* = 4–9) and C6 (*n* = 3–8) mice. The orange and blue horizontal lines represent the mean frequencies of RORγt^+^ Helios^−^ Tregs from the SPF and GF cohorts, respectively (from Figure 2E). All mice were 10–13 weeks old. SCFA and RORγt^+^Helios^−^ Treg data are pooled from at least two independent experiments. Each symbol represents an individual mouse. Black horizontal lines depict the geometric mean **(B)** or mean **(C,E)**. ***p < 0.001, ****p < 0.0001, not significant (ns), calculated by one-way ANOVA with Tukey's **(B,E)** or Dunnett's **(C)** post-test.

Since they are often obligate anaerobes, many Clostridiales species are unable to monocolonize a germ-free gut. We therefore added *B. coccoides* YL58 to C2 to generate community C5 and added *C. clostridioforme* YL32 to C5 to generate C6 (Supplementary Table 1). Successful addition of these species was confirmed by 16S rRNA amplicon sequencing (Figure 4A). We found that total IgE levels were not altered from germ-free levels in either C5- or C6-colonized mice (Figure 4B). Addition of the Clostridiales species also did not significantly alter SCFA levels compared to C4-colonized mice (Figure 4C), despite induction of RORγt^+^Helios^−^ pTregs in this cohort (Figures 4D,E and Supplementary Figure 4). These results indicated that other Oligo-MM^12^ members are likely contributing to suppression of hyper-IgE.

### Acetate Produced by *B. coccoides* YL58 Contributes to Suppression of Serum IgE Levels

**Figure 5:**
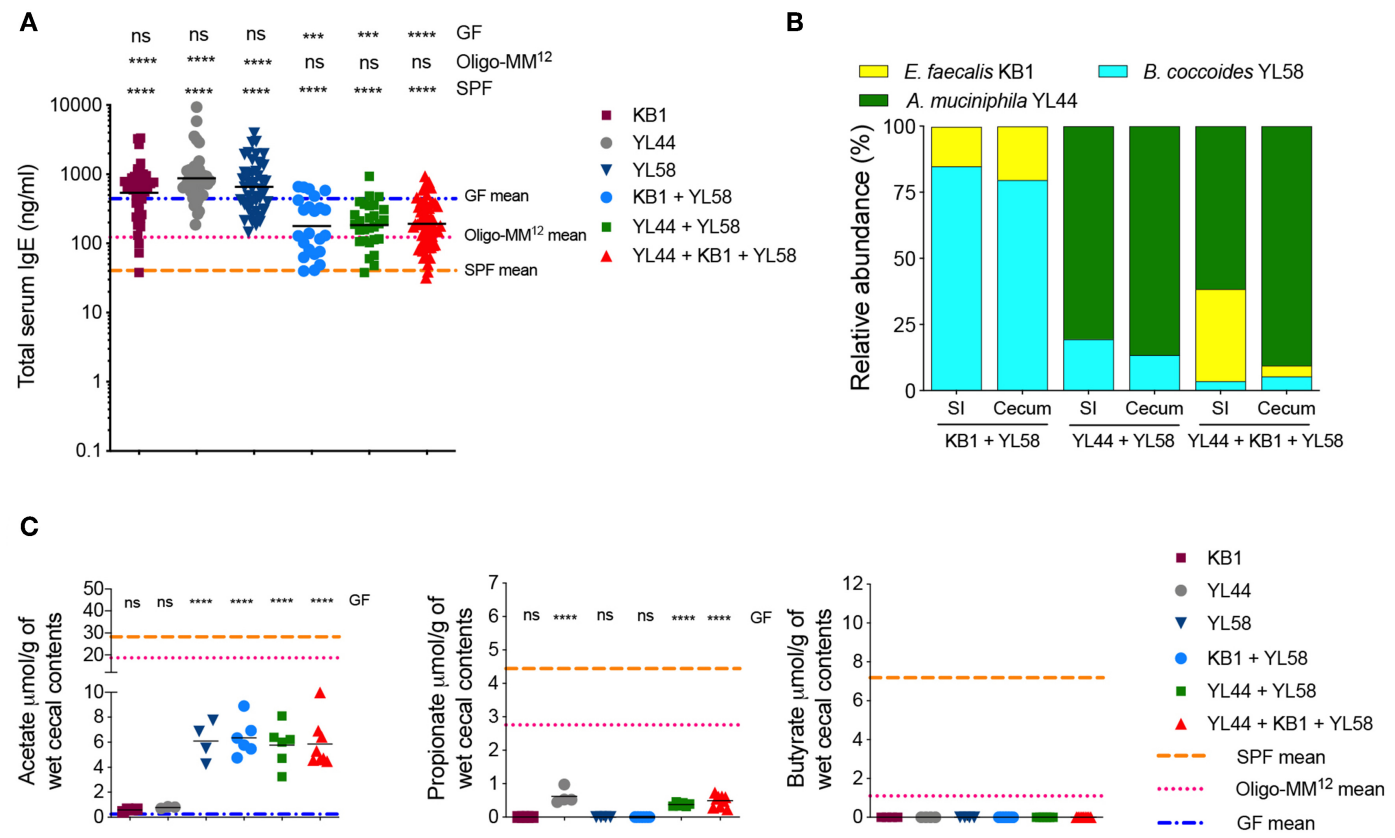
Cooperation between an immunogenic and an acetate-producing species can suppress hyper-IgE. **(A)** Total serum IgE levels in *E. faecalis* KB1 (*n* = 40), *A. muciniphila* YL44 (*n* = 40), *B. caecimuris* YL58 (*n* = 46), KB1 + YL58 (*n* = 23), YL44 + YL58 (*n* = 28), and KB1 + YL44 + YL58 (*n* = 61) colonized mice. The blue, pink, and orange horizontal lines represent the geometric mean of the serum IgE levels from the GF, Oligo-MM^12^ and SPF mice, respectively (from Figures 1B, 3B). **(B)** Relative species abundance in the small intestine (SI) and cecum of a representative KB1 + YL58, YL44 + YL58 and KB1 + YL44 + YL58 mouse, *n* = 3–4 mice per group. **(C)** SCFA levels in the cecal contents of KB1 (*n* = 4), YL44 (*n* = 3–4), YL58 (*n* = 4), KB1 + YL58 (*n* = 6), YL44 + YL58 (*n* = 6), and KB1 + YL44 + YL58 (*n* = 8–9) colonized mice. The blue, pink, and orange horizontal lines represent the mean SCFA levels from the GF, Oligo-MM^12^ and SPF cohorts, respectively (from Figures 1C, 3C). All mice were 10–13 weeks old. SCFA data are pooled from at least two independent experiments. Each symbol represents an individual mouse. Black horizontal lines depict the geometric mean **(A)** or mean **(C)**. ***p < 0.001, ****p < 0.0001, not significant (ns), calculated by one-way ANOVA with Tukey's post-test **(A)** or Dunnett's **(C)** post-test.

Since regulation of IgE is dependent on early-life microbial colonization (11), we next investigated whether *E. faecalis* KB1 and *A. muciniphila* YL44, the two most dominant members of the Oligo-MM^12^ consortia in early life (Figure 3F), were able to individually regulate serum IgE levels. We monocolonized breeding pairs with either *E. faecalis* KB1 or *A. muciniphila* YL44 and determined the serum IgE levels and cecal content SCFA concentrations in their offspring (Figure 5). Successful colonization with these individual bacteria was confirmed by 16S rRNA full length sequencing of DNA extracted from the feces and by gram staining of intestinal contents (data not shown). We found that total serum IgE levels in *E. faecalis* KB1 and *A. muciniphila* YL44 monocolonized mice were not significantly altered from germ-free levels (Figure 5A).

As neither *E. faecalis* KB1 nor *A. muciniphila* YL44 by themselves were able to suppress IgE we next tested these two in combination. Unfortunately, 16S rRNA amplicon sequencing of our double-colonized colony revealed that it got contaminated with the additional Oligo-MM^12^ species *B. coccoides* YL58 (YL44 + KB1 + YL58) (Figure 5B). We did, however, observe a reduction in serum IgE levels in this YL44 + KB1 + YL58 colony (Figure 5A). To investigate whether this effect relied on the presence of *B. coccoides* YL58 we generated *B. coccoides* YL58 monocolonized as well as *E. faecalis* KB1/*B. coccoides* YL58 (KB1 + YL58) and *A. muciniphila* YL44/*B. coccoides* YL58 (YL44 + YL58) double colonized cohorts. We found that while *B. coccoides* YL58 alone was not able to reduce serum IgE levels, we observed reduced IgE levels when *B. coccoides* YL58 was present in combination with *E. faecalis* KB1 or *A. muciniphila* YL44 (Figure 5A). This indicated that functional cooperation between bacterial species with different characteristics was required to induce an IgE-suppressing effect. Of note, pTreg proportions in these different cohorts did not correlate with reduced serum IgE levels indicating that inhibition of IgE was not mediated solely by induction of pTregs (Supplementary Figures 5, 6). In addition, the absence of butyrate in all cohorts (Figure 5C), and the absence of propionate in the KB1 + YL58 model suggests that propionate and butyrate are not required for suppressing hygiene-induced hyper-IgE. In contrast, acetate production correlated with reduced serum IgE levels with the exception of the *B. coccoides* YL58 monocolonized group (Figures 5B,C). Therefore, since *B. coccoides* YL58 alone was not sufficient to suppress IgE, we concluded that acetate provided by *B. coccoides* YL58 (Figure 5C) is required but not sufficient for reducing IgE levels. Since *E. faecalis* KB1 and *A. muciniphila* YL44 do not produce high levels of acetate (Figure 5C), we next investigated what could be the particular characteristics of *E. faecalis* KB1 and *A. muciniphila* YL44 that are required to suppress IgE when present in combination with an acetate-producer.

### Immunogenicity Is an Additional Requirement for IgE Suppression

A recent study identified *A. muciniphila* as a strong inducer of systemic IgA and IgG1 (48). We therefore measured serum IgA and IgG1 reactivity against *A. muciniphila* YL44 in the YL44 monocolonized, YL44 + YL58 bicolonized and YL44 + KB1 + YL58 tricolonized cohorts (Supplementary Figure 7). All *A. muciniphila* YL44 colonized cohorts displayed IgA and IgG1 reactivity against *A. muciniphila* YL44. While *E. faecalis* KB1 monocolonized mice also displayed specific serum IgA reactivity, *B. coccoides* YL58 monocolonized mice did not (Supplementary Figure 7). In order to extend this immunogenicity screen, we measured total IgA (as opposed to bacteria-specific IgA) in small intestinal washes and serum from various gnotobiotic cohorts (Supplementary Figure 8). We also included segmented filamentous bacteria (SFB) monocolonized mice as a control as SFB is known to be a potent inducer of IgA (49). We found that *A. muciniphila* YL44, *E. faecalis* KB1 and SFB were able to promote induction of IgA (Supplementary Figure 8). Importantly, although SFB induced a strong IgA response (Supplementary Figure 8) as well as a RORγt^+^Helios^−^ pTreg response in the colon, SFB alone was not sufficient to suppress hygiene-induced hyper-IgE, possibly due to the lack of acetate production (Supplementary Figure 9). We therefore conclude that IgA immunogenicity in combination with acetate production is required to drive an IgE suppressive phenotype.

### IL10 Production Does Not Correlate With Protection From Hyper IgE

Lastly, we investigated whether IL10 production in the small intestine, which is required to maintain intestinal immune homeostasis (50), was required to inhibit hygiene-induced hyper-IgE. We found similar proportions of IL10^+^CD45^+^TCRβ^−^ cells in the small intestine of cohorts with high vs. low serum IgE levels indicating that small intestinal IL10 production alone is not sufficient to suppress hygiene-induced hyper-IgE (Supplementary Figure 10). In addition, SFB monocolonized mice, which had the highest proportion of IL10^+^ cells (Supplementary Figure 10) retained hygiene-induced hyper-IgE (Supplementary Figure 9).

In summary, we generated an extensive range of clearly defined and quality controlled gnotobiotic microbiotas *in vivo* in combination with read-outs for serum IgE levels, intestinal SCFA levels, Treg induction, IgA and IL10 production to identify characteristics of individual or communities of commensal bacterial species with the capacity to suppress the hyper-IgE syndrome observed in germ-free mice. We found that the IgA-inducing *A. muciniphila* YL44 and *E. faecalis* KB1 bacterial species, both dominant in the small intestine in early life, can cooperate with acetate-producing *B. coccoides* YL58 to inhibit hyper-IgE. Furthermore, this inhibitory effect was independent of propionate and butyrate production, frequencies of IL10^+^CD45^+^TCRβ^−^ cells in the small intestinal lamina propria and frequencies of RORγt^+^Helios^−^ pTregs in the intestinal tissues and GALT.

## Discussion

Elevated serum IgE levels in germ-free, but also in colonized immuno-deficient animals, has been a long-standing observation (8). We have also previously demonstrated that there is a critical period in early life at around the time of weaning whereby exposure to a diverse group of microbes is essential to prevent isotype switching to IgE (11). However, the mechanism by which this aberrant IgE induction is regulated following intestinal colonization and which commensal species are potent mediators of protection remains unknown. Here we show that colonization with *A. muciniphila* or *E. faecalis*, that are both predominantly present before weaning and induce IgA, in combination with the acetate-producing bacterial species *B. coccoides* YL58, results in partial protection from serum hyper-IgE. Surprisingly, this protection did not correlate with microbial induction of RORγt^+^Helios^−^ pTregs in the mucosal tissues, induction of IL10^+^CD45^+^TCRβ^−^ cells, or production of propionate or butyrate.

We identified induction of IgA and mucosa association as key characteristics of the bacteria that were involved in inhibition of IgE. Indeed, both *A. muciniphila* and *E. faecalis* are present near the epithelial surface of the small and large intestines (51, 52). Although *A. muciniphila* is known to have barrier-protective functions (53, 54), it is also a potent inducer of bacteria-specific systemic IgA and IgG1 responses (48). Furthermore, decreases in *A. muciniphila* have been associated with childhood atopy (55). This indicates that *A. muciniphila* is an immunogenic commensal species and this characteristic contributes to inhibition of hygiene-induced IgE. In humans, a strong serum IgG response against a common set of intestinal microbial antigens has been associated with protection against allergy development during childhood (56). It is also known that mucosa-association promotes an IgA response (57). Nevertheless, these characteristics are not sufficient to inhibit IgE because monocolonization with *A. muciniphila, E. faecalis* or SFB failed to reduce IgE levels.

Interestingly, we identified acetate production as a critical characteristic required for IgE inhibition. B cell class switch to IgE occurs in the PP in germ-free mice (11) and we found that acetate (1.9 ± 0.31 μmol/gram), but not butyrate or propionate, and the acetate-producing bacteria *B. coccoides* YL58 was present in the small intestine. In addition, ffar2/gpr43, an acetate receptor, is also expressed in the small intestine, predominantly on leukocytes in the lamina propria (58). In humans, acetate was found to be significantly reduced in infants at 3 months of age who subsequently developed atopy and wheeze (59) and acetate has also been identified to be the most abundant SCFA in the feces of 3–5-month-old infants (60). Therefore, acetate may be an important bacterial-derived metabolite that regulates early life immunity, including regulation of IgE.

We have previously shown that there is a critical window in early life where microbial colonization is required to inhibit hygiene-induced IgE (11). We now show that the bacteria that are capable of IgE inhibition are dominant in early life prior to weaning.

Al Nabhani et al. has recently characterized a vigorous immune response at weaning, termed the weaning reaction, as it is associated with changes in the microbiota at weaning (61). The weaning reaction consists of a time-dependent increase in tumor necrosis factor alpha (TNF-α) and interferon-γ (IFNγ), and is driven by bacteria, SCFA and vitamin A (61). Thus, it is tempting to speculate that a strong weaning reaction induced by bacteria such as *A. muciniphila* YL44 and *E. faecalis* KB1 in the presence of an acetate-producing bacteria (such as *B. coccoides* YL58) during this critical window is sufficient to suppress IgE. This colonization status could potentially favor IgA and IgG1 isotype class switching events while still inducing the important RORγt^+^Helios^−^pTregs population (61). Although induction of RORγt^+^Helios^−^ pTregs at weaning was critical for protection from inflammation later in life (61), our data indicates that induction of RORγt^+^Helios^−^ pTregs alone is insufficient to inhibit IgE.

Taken together, we show that both bacterial richness and the presence of key bacterial species in early life are important for regulating serum IgE. A recent publication has demonstrated that the increased IgE levels in germ-free mice are induced by food antigens (62). In context of the weaning reaction (61) and our findings, this suggests that appropriate bacterial signals early in life are required to prevent sensitization to food antigens. Therefore, in addition to the Clostridia species previously identified (63), our data provides additional bacterial candidates and avenues for microbial-based prevention strategies in food allergy.

It is important to note that our bacterial combinations only provided partial suppression of serum IgE levels and other additional microbial characteristics are needed to fully inhibit IgE to the levels found in SPF mice. Therefore, it will be interesting to further investigate the role of other bacterial characteristics and metabolites on IgE regulation in order to develop microbial-based therapeutics for prevention of allergic diseases in humans. Furthermore, it would be interesting to screen for commensal species that are dominant in early life, induce IgA and produce acetate, to see whether these species alone can suppress hyper-IgE. Unfortunately, thus far we did not come across species with these capabilities in our extensive *in vivo* gnotobiotic screen. Alternatively, once appropriate genetic tools are available, *A. muciniphila* YL44 and *E. faecalis* KB1 can be genetically modified to produce acetate and tested in isolation.

## Data Availability Statement

The 16S rRNA amplicon sequences generated for this study can be found on the figshare repository at https://doi.org/10.6084/m9.figshare.c.4763306.

## Ethics Statement

The animal study was reviewed and approved by The Commission for Animal Experimentation of the Veterinary Office of Canton Bern and the Health Sciences Animal Care Committee of the University of Calgary.

## Author Contributions

MW contributed to the experimental design, performed and analyzed all the experiments, and contributed to writing the manuscript. KB contributed to the bioinformatics analysis. FT, KB, CT, and FR contributed to experiments assessing pTreg induction. MK and VF performed and analyzed the bacterial FACS. DB and IL established the targeted metabolomic protocol for the SCFAs. MG and KM analyzed and interpreted the data and wrote the manuscript. KM conceived and designed the project.

### Conflict of Interest

The authors declare that the research was conducted in the absence of any commercial or financial relationships that could be construed as a potential conflict of interest.
