# Supplementary material for: Using Precisely Defined in vivo Microbiotas to Understand Microbial Regulation of IgE
Source: Front Immunol. 2020 Jan 15;10:3107. doi: 10.3389/fimmu.2019.03107 (PMC6974480; doi:10.3389/fimmu.2019.03107)
Supplement: Supplementary file 1 [file Data_Sheet_1.PDF]

Supplementary Figure 1

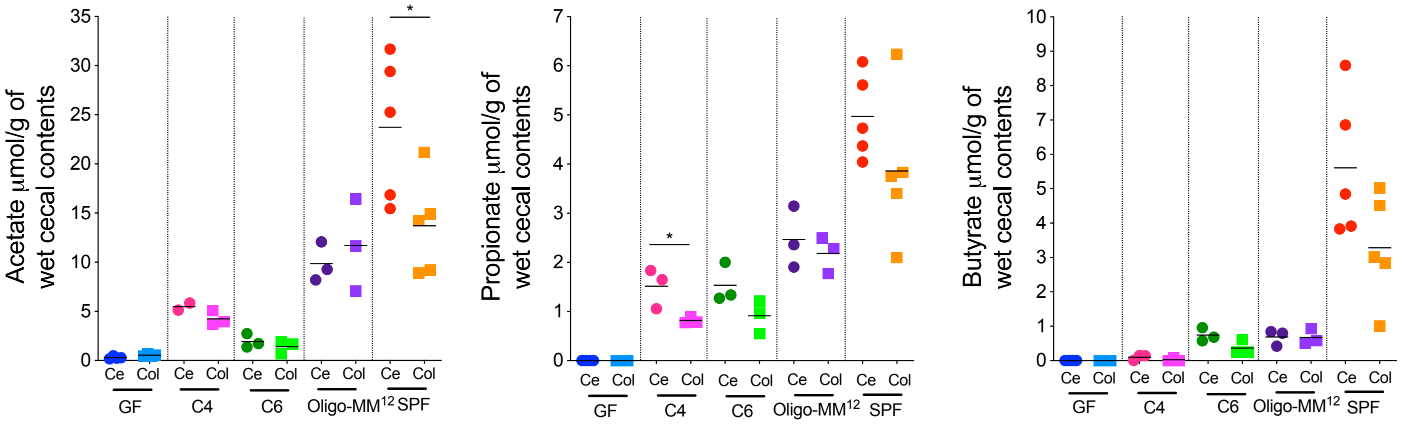

Supplementary Figure 1. Comparison of SCFA concentrations in the cecal and colon contents of the same GF, C4, C6, Oligo-MM^{12} and SPF mice, *n*=3-5 per group. All mice were between 10 to 14 weeks old. Each symbol represents an individual mouse. Black horizontal lines depict the mean. *p<0.05 calculated by Student's t-test.

Supplementary Figure 2

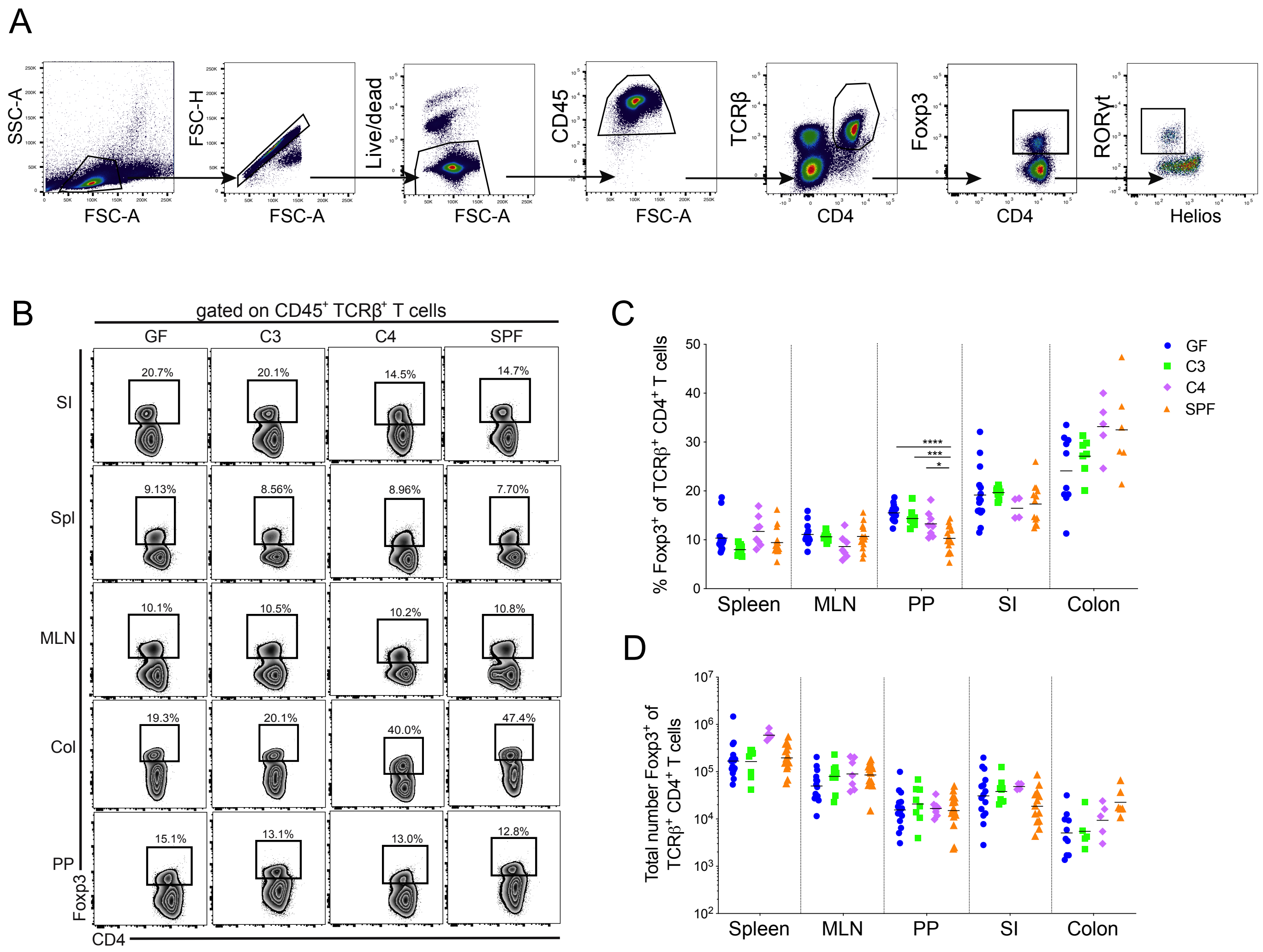

Supplementary Figure 2. Gating strategy for RORγt$^+$Helios$^-$ Tregs and frequencies and total numbers of Foxp3$^+$ T cells in the tissues of GF, C3, C4 and SPF mice. **(A)** Gating strategy for RORγt$^+$Helios$^-$ Tregs **(B)** Representative flow cytometry plots of Foxp3$^+$ T cells in different tissues (Spl, spleen; PP, Peyer's patches; MLN, mesenteric lymph nodes; SI, small intestinal lamina propria; Col, colon lamina propria) of GF, C3, C4 and SPF mice, **(C)** Frequencies and **(D)** total numbers of Foxp3$^+$ T cells among CD45$^+$TCRβ$^+$CD4$^+$ T cells in different tissues of GF (*n*=10-15), C3 (*n*=7-9), C4 (*n*=4-8) and SPF (*n*=6-16) mice. All mice were 10 to 13 weeks old. Data are pooled from at least two independent experiments. Each symbol represents an individual mouse. Black horizontal lines depict the mean. *p<0.05, **p<0.05, ***p<0.001, ****p<0.0001, calculated by one-way ANOVA with Tukey's post-test.

Supplementary Figure 3

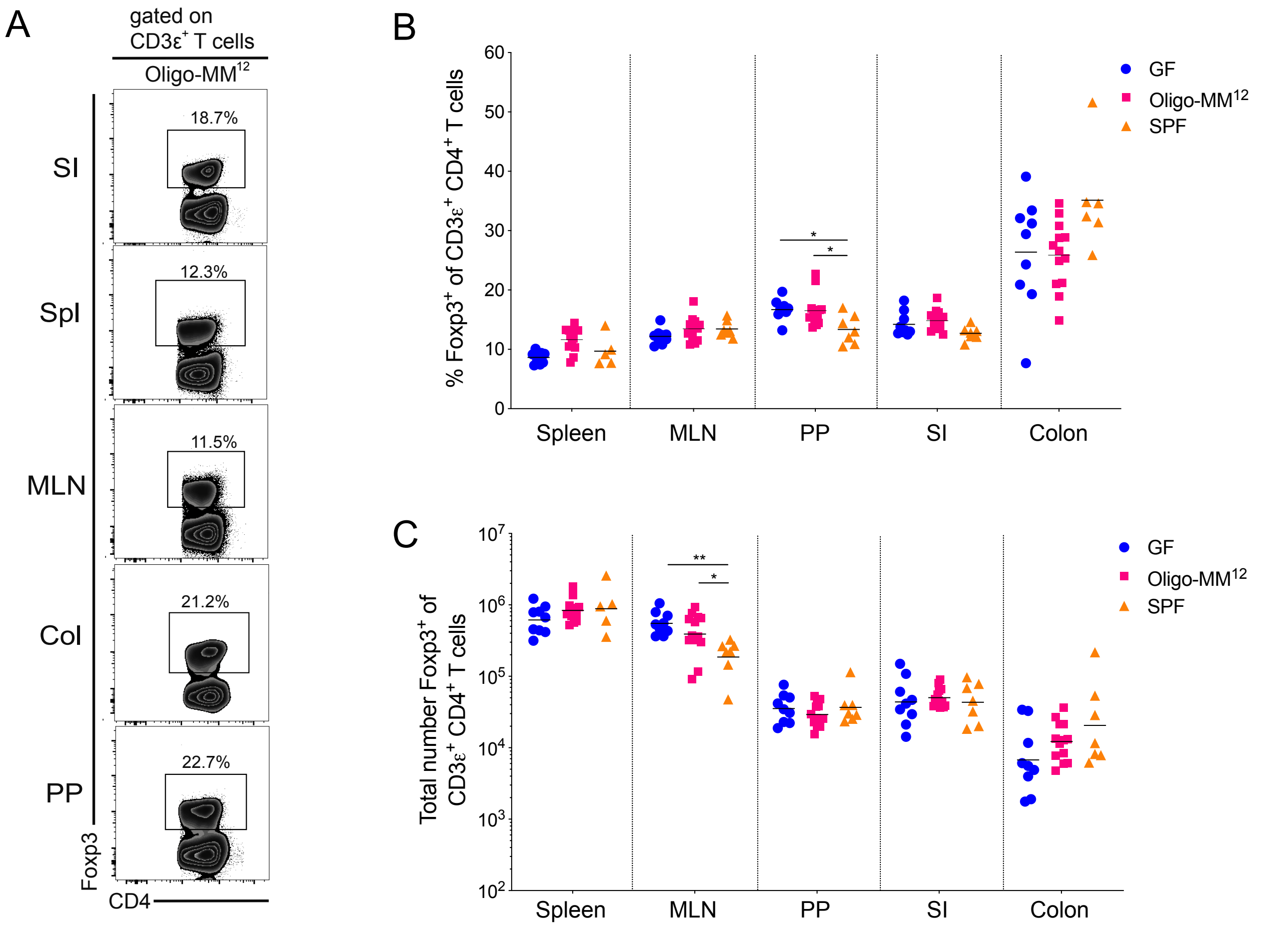

Supplementary Figure 3. Frequencies and total numbers of Foxp3$^+$ T cells in the tissues of Oligo-MM^{12} mice. **(A)** Representative flow cytometry plots of Foxp3$^+$ T cells among CD3ε$^+$CD4$^+$ T cells in different tissues (Spl, spleen; PP, Peyer's patches; MLN, mesenteric lymph nodes; SI, small intestinal lamina propria; Col, colon lamina propria) of Oligo-MM^{12} mice. **(B)** Frequencies and **(C)** total numbers of Foxp3$^+$ T cells in different tissues of GF (*n*=9) , Oligo-MM^{12} (*n*=12-13) and SPF (*n*=5-7) mice. All mice were 10 to 13 weeks old. Tregs data are pooled from at least two independent experiments. Each symbol represents an individual mouse. Black horizontal lines depict the mean. *p<0.05, **p<0.01, ***p<0.001, ****p<0.0001, calculated by one-way ANOVA with Tukey's post-test.

Supplementary Figure 4

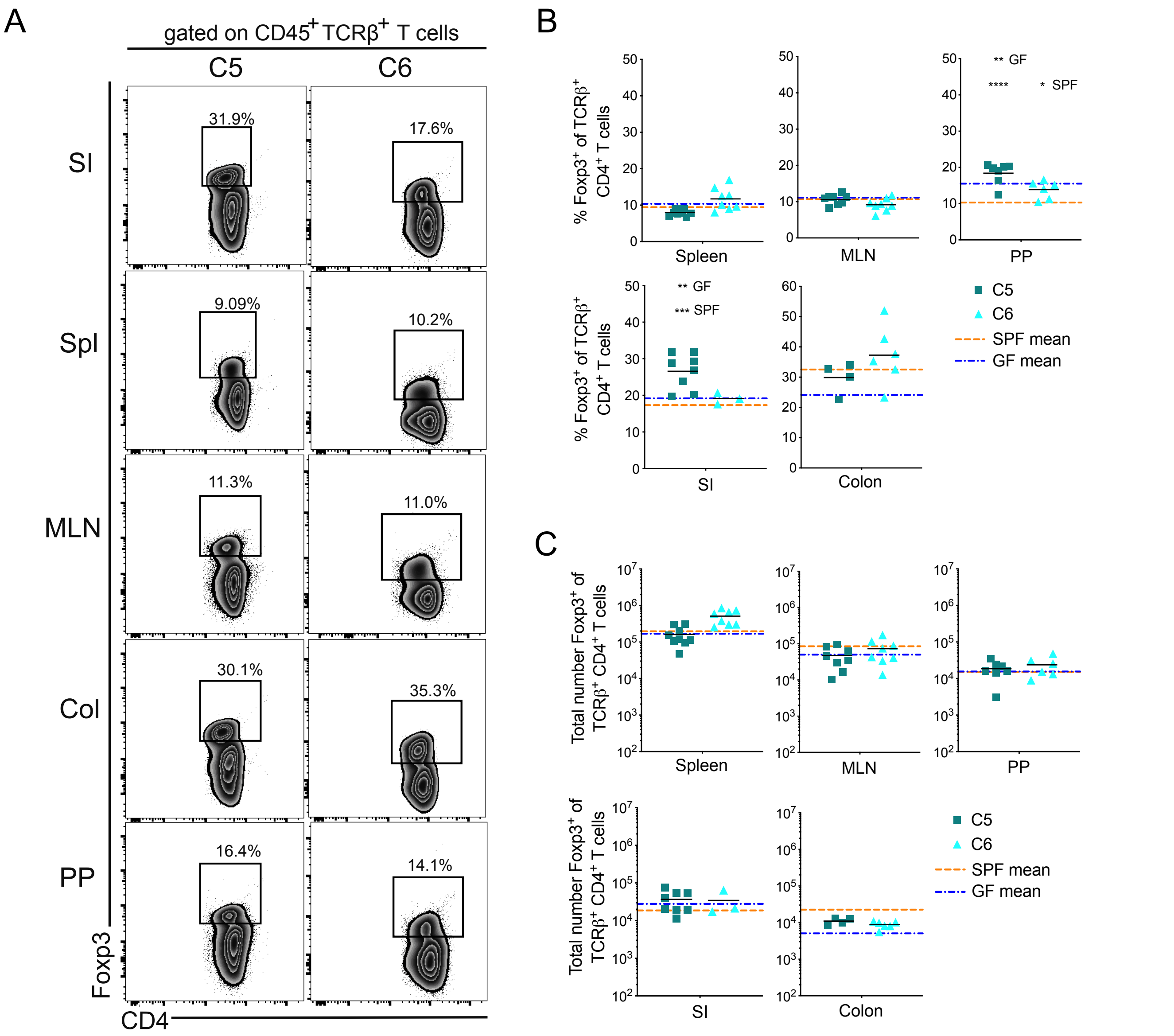

Supplementary Figure 4. Frequencies and total numbers of Foxp3+ T cells in the tissues of C5- and C6-colonized mice. **(A)** Representative flow cytometry plots of Foxp3+ T cells among CD45+TCRβ+CD4+ T cells in different tissues (Spl, spleen; PP, Peyer's patches; MLN, mesenteric lymph nodes; SI, small intestinal lamina propria; Col, colon lamina propria) of C5 and C6 mice. **(B)** Frequencies and **(C)** total numbers of Foxp3+ T cells in different tissues of C5 (*n*=4-9) and C6 (*n*=3-8) mice. The blue and orange horizontal lines represent the mean frequencies or total numbers of the Foxp3+ T cell populations from the GF and SPF cohorts, respectively (from Supplementary Figure 2C and 2D). All mice were 10 to 13 weeks old. Tregs data are pooled from at least two independent experiments. Each symbol represents an individual mouse. Black horizontal lines depict the mean. *p<0.05, **p<0.01, ***p<0.001, ****p<0.0001, calculated by one-way ANOVA with Tukey's post-test.

Supplementary Figure 5

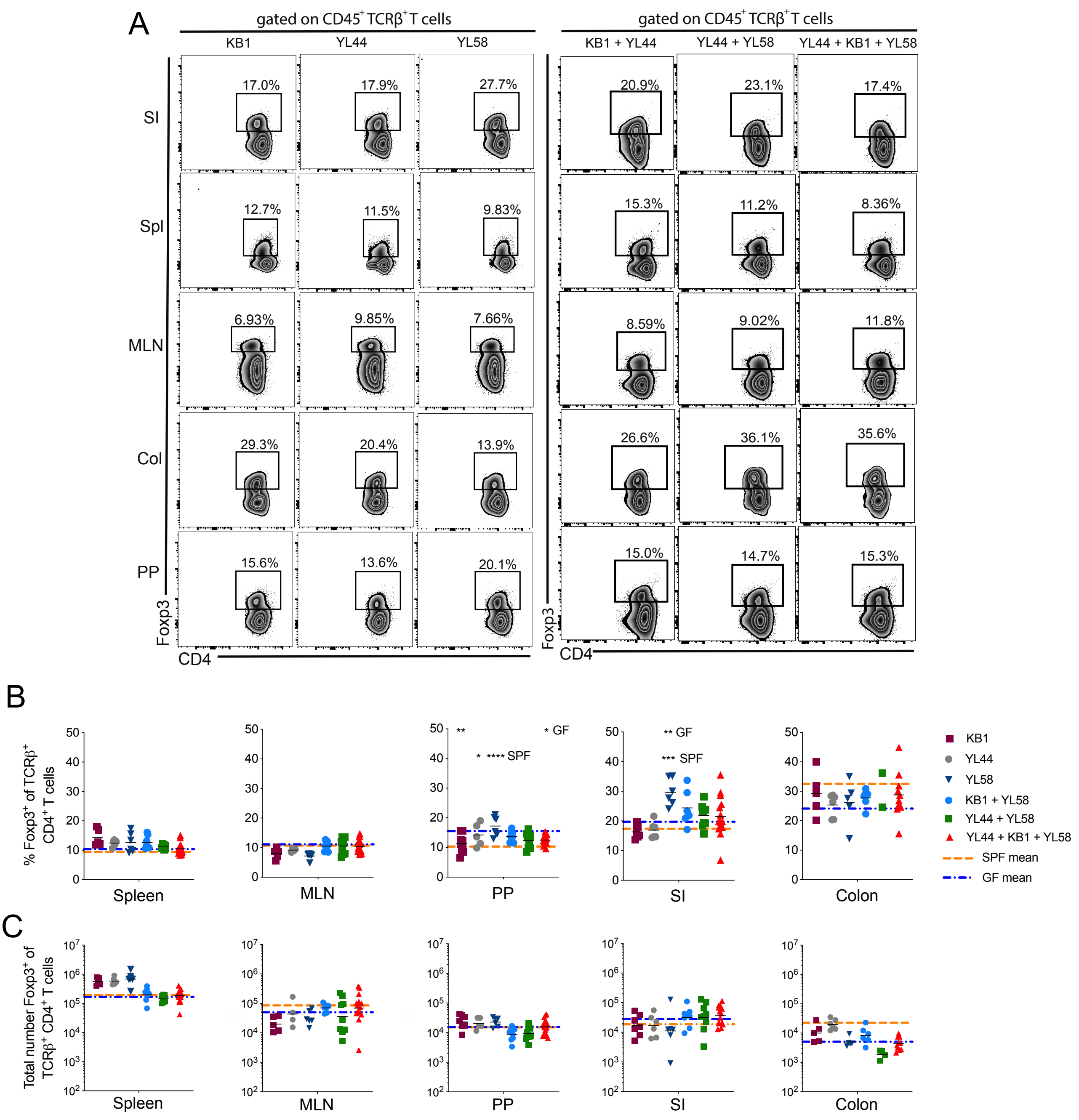

Supplementary Figure 5. Frequencies and total numbers of Foxp3$^+$ T cells in the tissues of six gnotobiotic mouse models. **(A)** Representative flow cytometry plots of Foxp3$^+$ T cells among CD45$^+$TCRβ$^+$CD4$^+$ T cells in different tissues (Spl, spleen; PP, Peyer's patches; MLN, mesenteric lymph nodes; SI, small intestinal lamina propria; Col, colon lamina propria) of *E. faecalis* KB1, *A. muciniphila* YL44, *B. caecimuris* YL58 monocolonized mice and KB1 + YL58, YL44 + YL58 and KB1 + YL44 + YL58 colonized mice **(B)** Frequencies and **(C)** total numbers of Foxp3$^+$ T cells in different tissues of KB1 (*n*=5-7), YL44 (*n*=4-6), YL58 (*n*=5-6), KB1 + YL58 (*n*=6-8), YL44 + YL58 (*n*=4-9) and KB1 + YL44 + YL58 (*n*=10-16) mice. The blue and orange horizontal lines represent the mean frequencies or total numbers of the Foxp3$^+$ T cell populations of the GF and SPF cohorts, respectively (from Supplementary Figure 2C and 2D). All mice were 10 to 13 weeks old. Tregs data are pooled from at least two independent experiments. Each symbol represents an individual mouse. The black horizontal lines depict the mean. *$p<0.05$, **$p<0.01$, ***$p<0.001$, ****$p<0.0001$, calculated by one-way ANOVA with Tukey's post-test.

Supplementary Figure 6

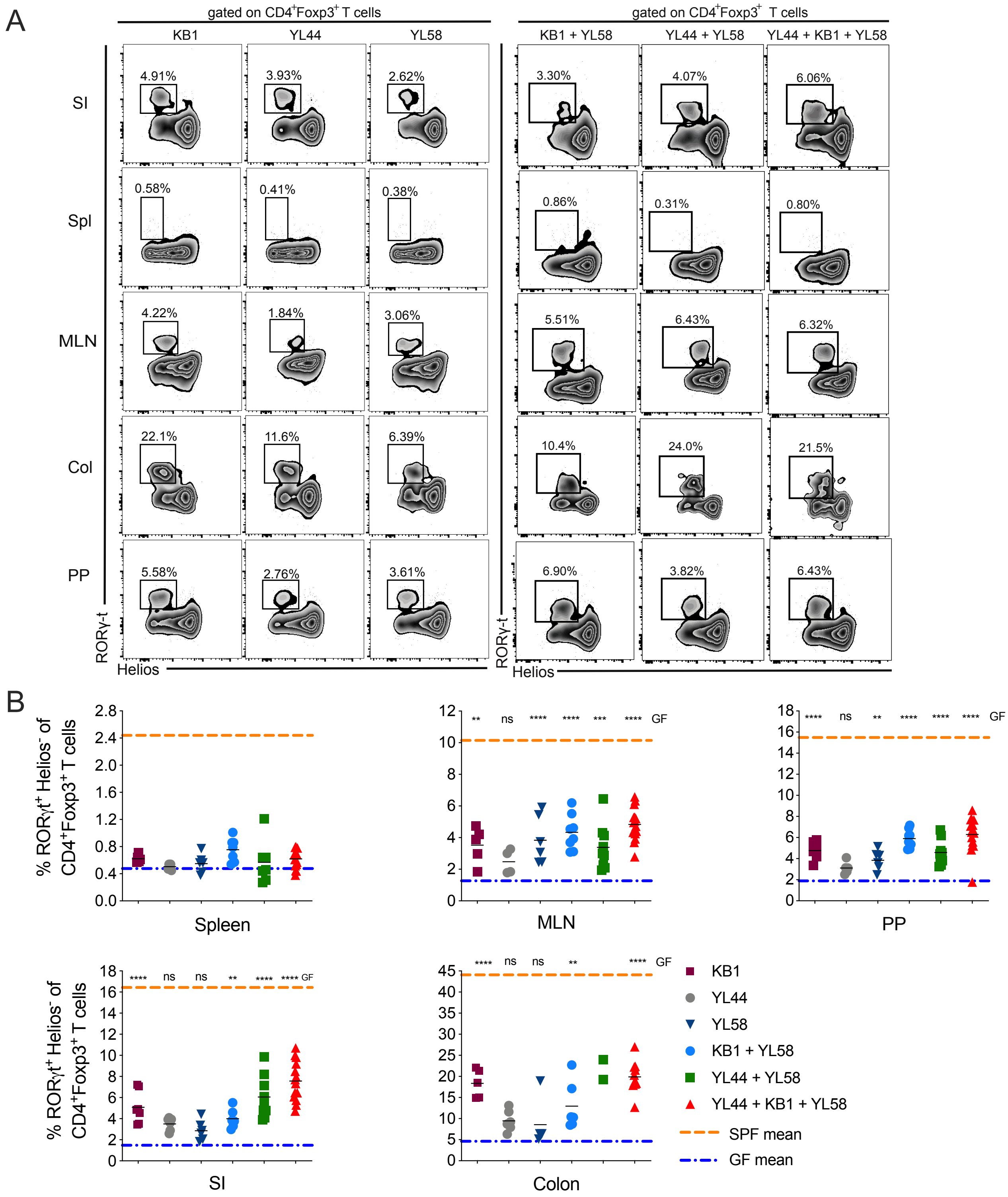

Figure 6. pTreg frequencies do not correlate with the capacity to suppress hyper-IgE.
(A) Representative flow cytometry plots of RORγt$^+$Helios$^-$ Tregs in different tissues (Spl, spleen; PP, Peyer's patches; MLN, mesenteric lymph nodes; SI, small intestinal lamina propria; Col, colon lamina propria) of *E. faecalis* KB1, *A. muciniphila* YL44, *B. caecimuris* YL58 monocolonized, KB1 + YL58, YL44 + YL58 and KB1 + YL44 + YL58 mice. **(B)** Frequencies of RORγt$^+$Helios$^-$ Tregs among CD45$^+$TCRβ$^+$CD4$^+$Foxp3$^+$ T cells in different tissues of KB1 (*n*=5-7), YL44 (*n*=4-6), YL58 (*n*=5-6), KB1 + YL58 (*n*=6-8), YL44 + YL58 (*n*=2-9) and KB1 + YL44 + YL58 (*n*=10-16) mice. The blue and orange horizontal lines represent the mean frequencies of RORγt$^+$ Helios$^-$ Tregs from the GF and SPF cohorts, respectively (from Figure 2E). All mice were 10 to 13 weeks old. Data are pooled from at least two independent experiments. Each symbol represents an individual mouse. Black horizontal lines depict or mean. *$p<0.05$, **$p<0.01$, ***$p<0.001$, ****$p<0.0001$, not significant (ns), calculated by one-way ANOVA with Tukey's post-test.

Supplementary Figure 7

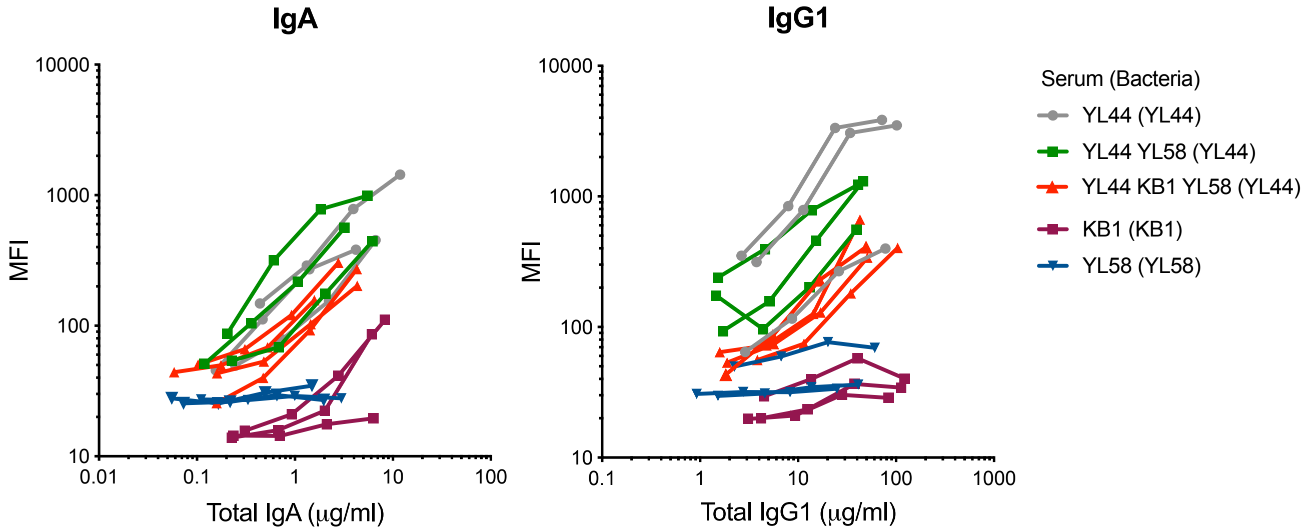

Supplementary Figure 7. Bacterial flow cytometric analysis with serial dilutions of serum from mice monocolonized with *A. muciniphila* YL44 or *E. faecalis* KB1 or *B. coccoides* YL58 and mice colonized with either YL44 + YL58 or YL44 + KB1 + YL58. The bacterial species the serum was incubated with is indicated in parentheses. Each line represents one mouse, *n*=3-4 mice per group. One representative of three independent experiments is shown.

Supplementary Figure 8

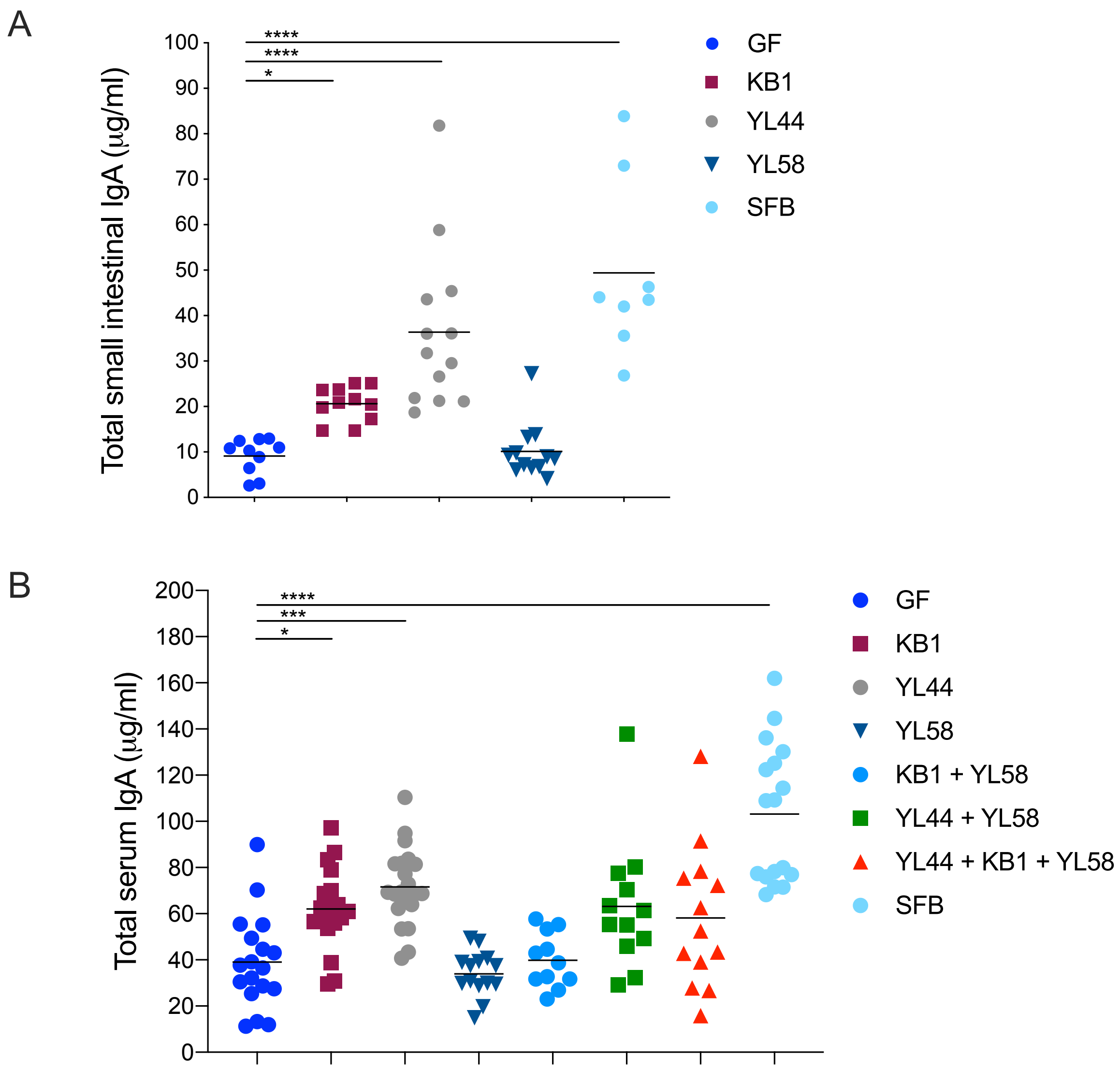

Supplementary Figure 8. SFB, *A. muciniphila* YL44 and *E. faecalis* KB1 can induce a strong IgA response in the small intestine and serum. **(A)** IgA concentrations in the small intestine and **(B)** serum of mice under different hygiene conditions. *n*=8-21 mice per group. All mice were 10 to 14 weeks old. Each symbol represents an individual mouse. Black horizontal lines depict the mean. *p<0.05, **p<0.01, ***p<0.001, ****p<0.0001, calculated by Kruskal-Wallis with Dunn's post-test.

Supplementary Figure 9

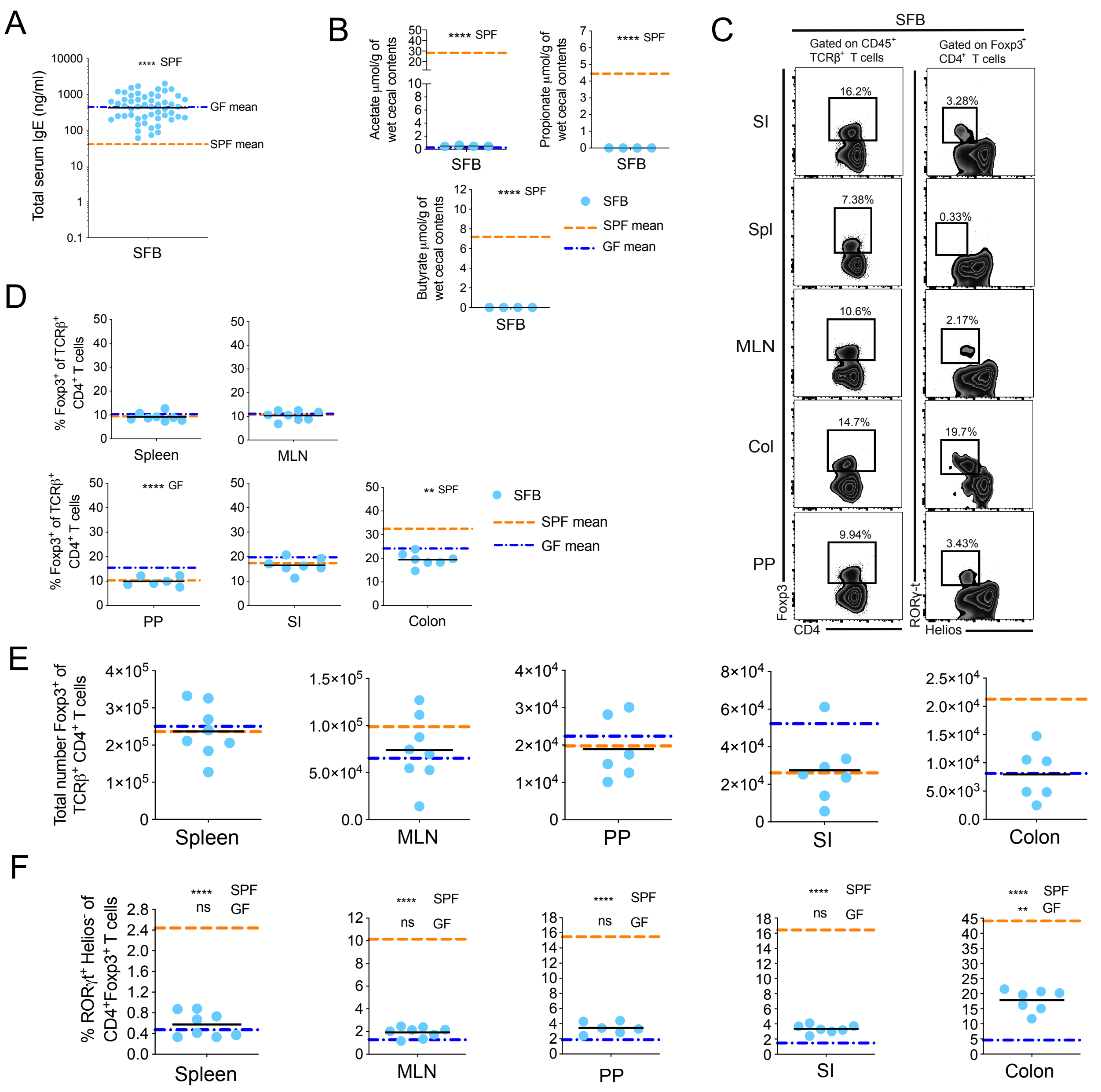

Supplementary Figure 9. SFB monocolonization cannot inhibit hyper-IgE. **(A)** Total serum IgE levels in SFB monocolonized mice (*n*=53). The blue and orange horizontal lines represent the geometric mean of the serum IgE levels from GF and SPF cohorts, respectively (from Figure 1B). **(B)** SCFA levels in the cecal contents of SFB mice (*n*=4). The blue and orange horizontal lines represent the mean SCFA levels from the GF and SPF cohorts, respectively (from Figure 1C). **(C)** Representative flow cytometry plots of Foxp3$^+$ T cells among CD45$^+$TCRβ$^+$CD4$^+$ T cells and RORγt$^+$Helios$^-$ Tregs in different tissues (Spl, spleen; PP, Peyer's patches; MLN, mesenteric lymph nodes; SI, small intestine lamina propria; Col, colon lamina propria) of SFB mice. **(D)** Frequencies and **(E)** total numbers of Foxp3$^+$ T cells among CD45$^+$TCRβ$^+$CD4$^+$ T cells in different tissues of SFB mice (*n*=6-8). **(F)** Frequencies of RORγt$^+$Helios$^-$ Tregs among CD45$^+$TCRβ$^+$CD4$^+$Foxp3$^+$ T cells in different tissues of SFB mice. The blue and orange horizontal lines represent the respective means from the GF and SPF cohorts' T cell populations, respectively (from Supplementary Figure 2C and 2D and Figure 2E). All mice were 10 to 13 weeks old. Tregs and RORγt$^+$Helios$^-$ Tregs data are pooled from at least two independent experiments. Each symbol represents an individual mouse. The black horizontal lines depict the geometric mean (A) or mean (B, D, E and F). *p<0.05, **p<0.01, ***p<0.001, ****p<0.0001, calculated by one-way ANOVA with Tukey's post-test (A, D, E and F) or with Dunnett's post-test (B).

Supplementary Figure 10

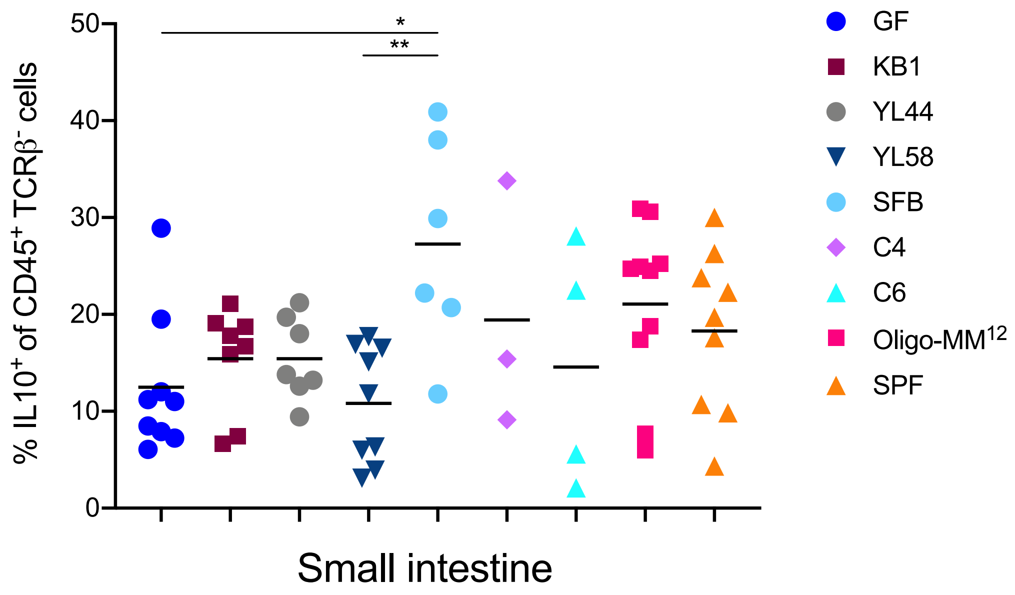

Supplementary Figure 10. Frequencies of IL10$^+$ cells of CD45$^+$ TCRβ$^-$ cells in the small intestine of mice under different hygiene conditions. *n*=3-10 mice per group. All mice were between 11 to 14 weeks old. Each symbol represents an individual mouse. Black horizontal lines depict the mean. Data is pooled from three independent experiments. *p<0.05, **p<0.01. ***p<0.001, ****p<0.0001, calculated by one-way ANOVA with Tukey's post-test.

Supplementary Table 1. Composition of the gnotobiotic communities generated for this study.

Gnotobiotic model	Composition	Related to Figure
Community 1 (C1)	*Lactobacillus murinus* ASF361<br>*Lactobacillus intestinalis* ASF360<br>*Parabacteroides distasonis* ASF519	Fig. 1
Community 2 (C2)	C1 + *Pseudoflavonifractor* sp. ASF500	Fig. 1
Community 3 (C3)	C2 + *Clostridia* sp. ASF356	Fig. 2
Community 4 (C4)	C3 + *Clostridia* sp. ASF502	Fig. 2
Community 5 (C5)	C2 + *Blautia coccoides* YL58	Fig. 4
Community 6 (C6)	C5 + *Clostridia clostridioforme* YL32	Fig. 4

Supplementary Table 2. ^{13}C Spiking concentrations of acetate, propionate and butyrate for cecal contents

Gnotobiotic model	^{13}C-Acetate (μM)	^{13}C-Propionate (μM)	^{13}C-Butyrate (μM)
C2	500	200	200
C3	500	400	200
C4	1000	400	200
C5	1000	400	200
C6	1000	400	200
Oligo-MM[12]	10000	500	500
SFB	1000	100	100
KB1	5000	100	100
YL44	5000	200	100
YL58	5000	100	100
KB1 + YL58	5000	200	100
YL44 + YL58	5000	200	100
YL44 + KB1 + YL58	5000	200	100
GF	100	10	10
SPF	10000	2000	3000

Supplementary Table 3. Composition of Oligo-MM^{12} mice

Gnotobiotic model	Composition	Related to Figure
Oligo-MM^{12} or sDMDMm2	*Clostridium innocuum* I46<br>*Bacteroides caecimuris* I48<br>*Lactobacillus reuteri* I49<br>*Enterococcus faecalis* KB1<br>*Acutalibacter muris* KB18<br>*Bifidobacterium animalis* subsp. *animalis* YL2<br>*Muribaculum intestinale* YL27<br>*Flavonifractor plautii* YL31<br>*Clostridium clostridioforme* YL32<br>*Akkermansia muciniphila* YL44<br>*Turicimonas muris* YL45<br>*Blautia coccoides* YL58	Fig. 3
